# New 1,2,3-Triazole Scaffold Schiff Bases as Potential Anti-COVID-19: Design, Synthesis, DFT-Molecular Docking, and Cytotoxicity Aspects

**DOI:** 10.3390/vaccines9091012

**Published:** 2021-09-11

**Authors:** Musa A. Said, Daoud J. O. Khan, Fawzia F. Al-blewi, Nadia S. Al-Kaff, Adeeb A. Ali, Nadjet Rezki, Mohamed Reda Aouad, Mohamed Hagar

**Affiliations:** 1 Department of Chemistry, College of Science, Taibah University, Al-Madinah Al-Munawarah 30002, Saudi Arabia; dnnnnnd@hotmail.com (D.J.O.K.); fbalawi@taibahu.edu.sa (F.F.A.-b.); aksheikhali@taibahu.edu.sa (A.A.A.); nrezki@taibahu.edu.sa (N.R.); 2 Department of Biology, College of Science, Taibah University, Al-Madinah Al-Munawarah 30002, Saudi Arabia; nkaff@taibahu.edu.sa; 3 Chemistry Department, College of Sciences, Taibah University, Yanbu 30799, Saudi Arabia; mhagar@taibahu.edu.sa; 4 Chemistry Department, Faculty of Science, Alexandria University, Alexandria 21321, Egypt

**Keywords:** 1,2,3-triazole, Schiff base (SB), DFT-QSAR, COVID-19, molecular docking, cytotoxicity

## Abstract

Schiff bases encompassing a 1,2,3-triazole motif were synthesized using an efficient multi-step synthesis. The formations of targeted Schiff base ligands were confirmed by different spectroscopic techniques (FT-IR, ^1^H NMR, ^13^C NMR, and CHN analysis). The spectral data analysis revealed that the newly designed hydrazones exist as a mixture of *trans-E* and *cis*-*E* diastereomers. Densityfunctional theory calculations (DFT) for the Schiff bases showed that the ***trans-trans*** form has the lowest energy structure with maximum stability compared to the other possible geometrical isomers that could be present due to the orientation of the amidic NH–C=O group. The energy differences between the ***trans***-***trans*** on one side and ***syn-syn*** and ***syn-trans*** isomers on the other side were 9.26 and 5.56 kcal/mol, respectively. A quantitative structure-activity relationship investigation was also performed in terms of density functional theory. The binding affinities of the newly synthesized bases are, maybe, attributed to the presence of hydrogen bonds together with many hydrophobic interactions between the ligands and the active amino acid residue of the receptor. The superposition of the inhibitor **N3** and an example ligand into the binding pocket of 7BQY is also presented. Further interesting comparative docking analyses were performed. Quantitative structure-activity relationship calculations are presented, illustrating possible inhibitory activity. Further computer-aided cytotoxicity analysis by Drug2Way and PASS online software was carried out for Schiff base ligands against various cancer cell lines. Overall, the results of this study suggest that these Schiff base derivatives may be considered for further investigation as possible therapeutic agents for COVID-19.

## 1. Introduction

The current design and discovery of new antiviral agents for confronting COVID-19 is engrossed mainly on attenuating the effects of the virus [1,2,3,4,5]. Thus, it has become urgently needful to develop new biochemical pharmacophores endowed with a broad spectrum of activity and less toxicity. Among many organic scaffolds, Schiff bases (SBs) possessing an azomethine linkage (R^1^–CH=N–R^2^) are well documented as promising and tunable molecules in drug discovery [6]. After the discovery of Schiff bases [7], millions of chemical structural variations were investigated. They were designed and developed to obtain the best chemotherapeutic results, especially for SBs incorporating a heterocyclic ring [8,9,10]. Due to their broad varying pharmacological activities, it was desirable to find efficient and effective synthesis methods, with continuing interest in organic synthesis. Several drugs containing this backbone are in clinical use, including muscle relaxants [11], antibiotics [12], antitubercular drugs [13], anti-inflammatory drugs [14], and antiviral agents [15]. In addition, among the significant class of nitrogen heterocyclic motifs, 1,2,3-triazoles have been the topic of substantial interest due to their broad spectrum of pharmacological uses and flexibility to modify their scaffolds for a particular biological application [16]. These tunable rings are being researched for drug evolution, as 1,2,3-triazoles can be structurally modified and investigated for their activity against several pharmacological targets to achieve desired molecules targeting a particular disease [17,18,19,20].

The 1,2,3-triazole cores can be found in many FDA-approved drugs such as rufinamide (anticonvulsant), TSAO (antiHIV), cefatrizine (antibiotic), tazobactam (antibacterial), CAI (anticancer), and ribavirin analogs (antiviral) [21,22,23,24]. This inspired us to synthesize new SBs clubbed to a 1,2,3-triazole core and chemically characterized using DFT calculations. Possible biological applications were also predicated by exploiting molecular docking and cytotoxicity, using computer-aided online software, as a continuation of our interest in developing novel bioactive heterocyclic-based SB linkages [25,26,27,28].

## 2. Results and Discussion

**Scheme 1:**
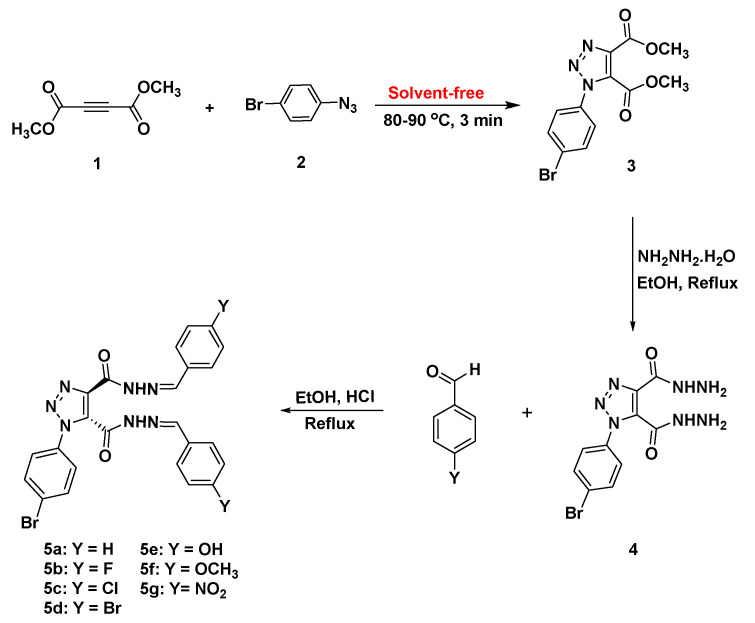
Synthesis of bis-acid hydrazide **4** and bis-Schiff bases bearing 1,2,3-triazole scaffold **5a**–**g**.

The newly designed SBs with a 1,2,3-triazole basic skeleton were prepared in a multi-step synthesis process as outlined in Scheme 1. Dimethyl 1-(4-bromophenyl)-1*H*-1,2,3-triazole-4,5-dicarboxylate (**3**) was successfully synthesized in 93% via solvent-free click coupling method of dimethylacetylene dicarboxylate (**1**) with *p*-bromoazidobenzene (**2**) for 3 min at 80–90 °C (Scheme 1). It should be noted that the azide **2** was prepared according to the reported procedure [29]. 

**Scheme 2:**
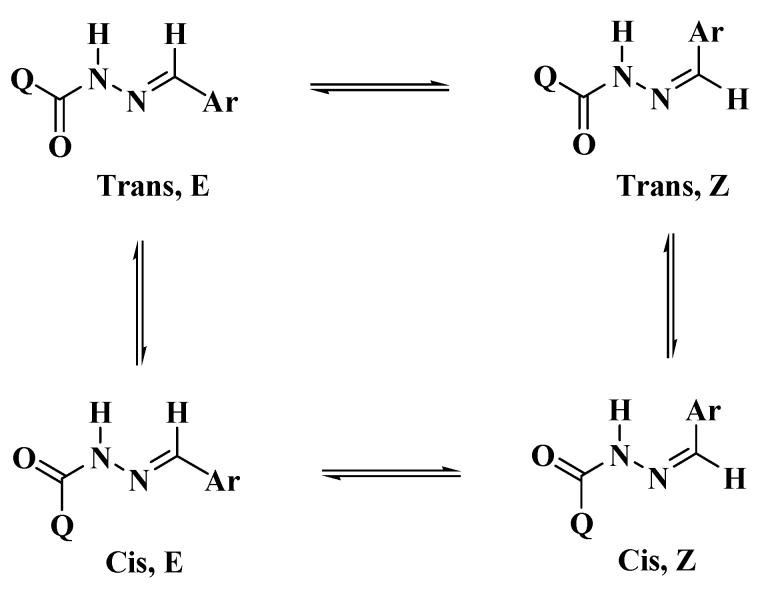
E/Z geometrical isomers and cis/trans conformers for hydrazones.

The targeted bis-hydrazones carrying 1,2,3-triazole **5a**–**g** were synthesized from the acid hydrazide precursor **4**via the thermal hydrazinolysis of bis-ester **3**. The condensation reaction needed reflux in ethanol for 4 h to afford the bis-acid hydrazide **4** in excellent yield (90%) (Scheme 1). The structures of the resulting 1,2,3-triazole-based ester and/or hydrazides **3**,**4** have been deduced based on their spectral data. The ^1^H-NMR spectrum of 1,2,3-triazole-diester **3** confirmed the success of the 1,3-dipolar cycloaddition reaction. The two nonequivalent methyl ester groups appeared as two distinct singlets at 3.88 and 3.94 ppm, respectively, which disappeared in the proton NMR spectrum of the corresponding acid hydrazide **4**. The spectrum of compound **4** also confirmed the presence of the characteristic hydrazide NH_2_ and NHprotonsas two broad singlets at 4.70 and 10.95 ppm, respectively. The aromatic phenyl protons were recorded as two doublets at 7.64 and 7.87 ppm. In the ^13^C-NMR spectrum of compound **4**, the absence of the two methyl ester carbons and the upfield shifting of the two ester carbonyl carbons (158.50 and 159.79 ppm) to the amide carbonyl carbons (155.45 and 158.50 ppm) confirmed the success of the hydrazinolysis reaction.1,2,3-Triazole-incorporated bis-acid hydrazide **4** was reacted with a series of un/substituted benzaldehydes in refluxing ethanol solution using a catalytic amount of HCl, as shown in Scheme 1. The resulting 1,2,3-triazoles bearing bis-Schiff base moieties **5a**–**g** were prepared in 87–90% yields. The ^1^H-NMR spectra of similar reported compounds showed the existence of E/Zgeometrical isomers in the imine (CH=N) bond and cis/trans conformers in the amide (C=O and N–H) group [30,31,32] (Scheme 2).

**Scheme 3:**
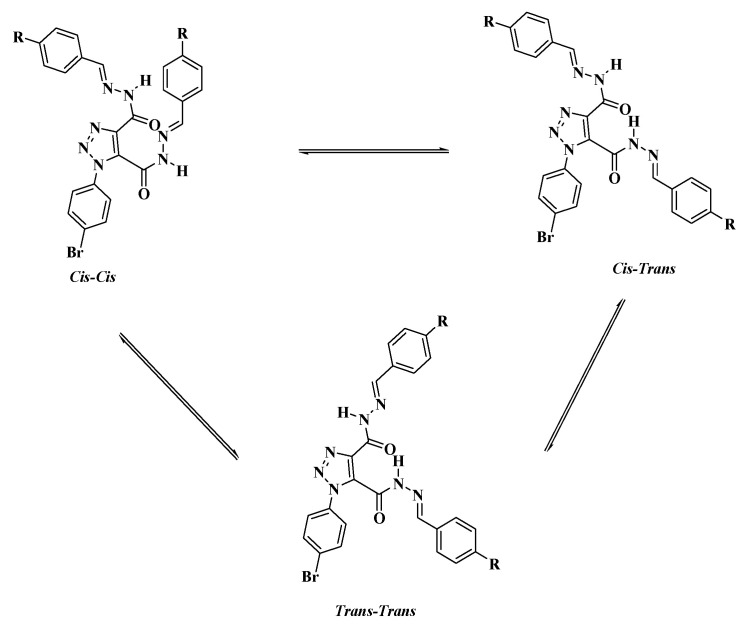
Possible conformational isomers for diacidhydrazones **5a**–**g**.

The *E* and *Z* geometrical isomers of the CH=N group were excluded from reports, which showed the E configuration of the C=N bond in DMSO solutions with steric hindrance on the imine bond, [33,34,35]. On the other hand, the Z isomer can be obtained only in a less polar solvent [36,37,38,39]. The molecular geometry of diacidhydrazones **5a**–**g** were assumed to exist in three conformational isomers, ***cis-cis***, ***trans-cis***, and ***trans-trans***, based on the orientation of the two amidic (C=O and N–H) groups, with respect to each other, of both arms of the bis-acid hydrazones (Scheme 3).

**Figure 1:**
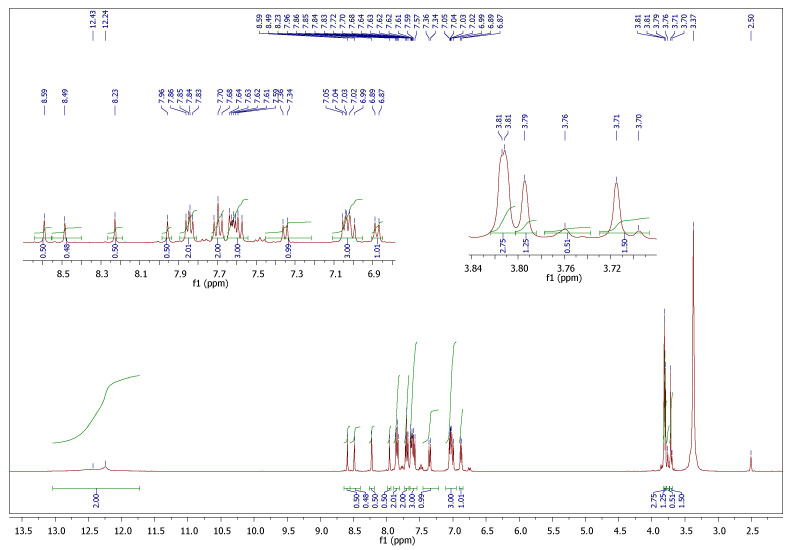
^1^H-NMR spectrum of compound **5f**.

Since we conducted our spectral analysis in DMSO-*d*_6_, two sets of isomers could be produced, i.e., the E/cis and the E/trans for each imino-amide moiety. The ^1^H and ^13^C NMR spectra for most of the compounds are provided in the supplementary materials file (Figures S1–S9). The ^1^H-NMR spectrum of the *p*-methoxy derivative **5f** (Figure 1) showed four different characteristic singlet peaks at 7.96, 8.23, 8.49, and 8.59 ppm at a ratio of 1:1:1:1 and integrated totally for two protons assigned to two imine protons (CH=N). Additionally, the two methoxy groups split into four singlets at 3.71, 3.76, 3.79, and 3.81 ppm and were integrated totally for six protons. The ^1^H-NMR spectrum also showed two broad singlets at 12.24 and 12.43 ppm integrated for the two NH groups and the aromatic protons. They appeared in their expected chemical shift and were integrated for two protons. The ^13^C-NMR spectrum (S9) confirmed the formation of the desired isomers through the appearance of three signals between 55.19–55.30 ppm for the corresponding methoxy groups. Furthermore, the four nonequivalent carbonyls (C=O) and imine (C=N) signals were observed at 152.93–161.25 ppm.

### 2.1. DFT Theoretical Calculations

#### Molecular Geometry

**Figure 2:**
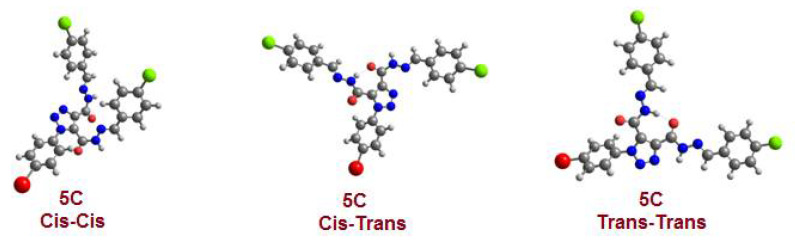
Calculated optimized molecular geometry of the proposed geometrical isomers of the chloro derivative **5c**.

In order to investigate the stability of the expected geometrical isomers, the optimized molecular structures were predicted by DFT calculations at the B3LYP 6-311G (d,p) basis set. The calculations were performed for the proposed three isomers of the prepared compound **5c** to predict the most stable geometrical isomer. These calculations involved carrying out a geometry structure optimization on each isomer to determine the minimum energy structure, followed by a frequency calculation at the optimized geometry. Various thermochemical quantities were also computed, Figure 2, Table 1.

To estimate the three geometrical isomers’ relative stabilities, the corrected energy, and the thermodynamic properties, enthalpy (H) and free energy (G) were calculated.

The results of the DFT calculations revealed that the ***trans-trans*** form has the lowest energy structure with maximum stability compared to the other geometrical isomers. However, the ***syn***-***syn*** isomer is the least stable. The energy difference between the ***trans***-***trans*** with the other isomers is 9.26 and 5.56 kcal/mol for ***syn***-***syn*** and ***syn***-***trans***, respectively. However, the ***trans-trans*** and ***syn-trans***’s higher stability could be illustrated in terms of the intramolecular H-bonding. On the other hand, the higher stability of the ***trans-trans*** when compared to the ***syn-trans*** could be explained in terms of the degree of H-bonding strength. The H-bonding strength was correlated to the length of the H-bond; it was 1.73 and 1.81 Å for the ***trans-trans*** and ***syn-trans***, respectively. The short H-bond of ***trans-trans*** could explain its higher stability.

**Figure 3:**
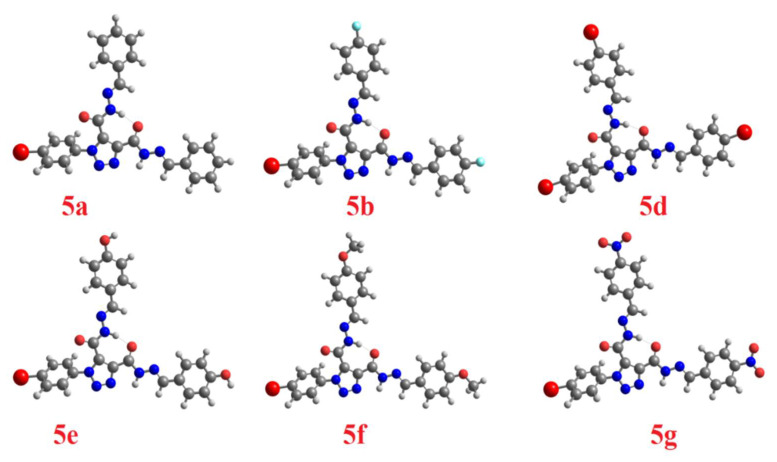
Calculated optimized molecular geometry of the ***trans***-***trans*** isomers of **5a**–**g**.

As we have previously reported, the electronic nature of the attached substituent to the arylidene part plays a significant role in stabilizing one geometrical isomer over the others where such groups’ polar nature could affect the strength of the intramolecular H-bonding. DFT has carried out the calculated optimized molecular structures of the ***trans-trans*** isomer for the other prepared derivatives at the same base set (Figure 3).

The effect of polar substituents on the stability of the ***trans***-***trans*** isomers has been related to the length of the H-bond. The predicted length of the H-bond was established by the theoretical calculations using the same method and the results have been tabulated in Table 2. As shown from the table, the order of the H-bond strength was **5a** < **5d** < **5e** < **5b** < **5g** < **5c** < **5f.**

### 2.2. Docking Study

**Figure 4:**
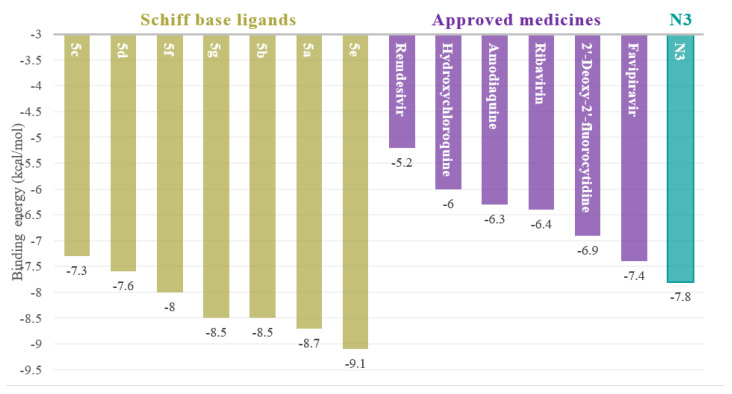
Binding affinities of SBs **5a**–**g** against COVID-19 7BQY protease (left). On the other side, approved medicines and ligand **N3** for comparison.

**Figure 5:**
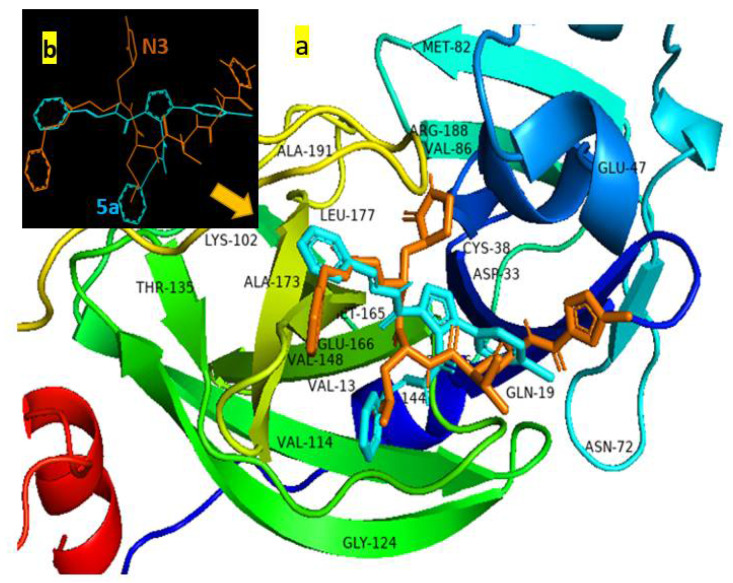
(**a**) The superposition of **N3** (orange stick) and compound **5a** (cyan stick) docked into the binding pocket of 7BQY represented by PyMOL [42]; (**b**)the superposition of **N3** (orange stick) and compound **5a** (cyan stick), where 7BQY is omitted for clarity.

A fair comparative study was conducted between the Schiff bases **5a**–**g** and U.S. FDA-approved antiviral drugs using 7BQY protease (**Resolution:** 1.7 Å) [40,41]. The binding affinities for the U.S. FDA-approved antiviral drugs against 7BQY protease were calculated using PyRx, AutoDock Vina, applying the same parameter as those used for computing the binding affinities for the SBs **5a**–**g** against7BQY protease in this study**.** binding affinities were detected for the SBs, ranging from –7.3 to –9.1 kcal/mol. However, the binding affinities of the approved antiviral drugs remdesivir, hydroxychloroquine, amodiaquine, ribavirin, 2′-Deoxy-2′-fluorocytidine, and favipiravir were in the range of −5.2 to −7.4 kcal/mol (Figure 4). The binding affinity of the reported inhibitor (**N3**), in nature, was −7.8 kcal/mol (obtained using the same parameters as for all samples in this study), shown in Figure 5.

A docked sample, **5a**, fitted in a cavity held by hydrogen bonding and a hydrophobic interaction with the active sites of protease 7BQY is presented in Figure 5a. The superposition of **N3** and compound **5a** after being docked into the binding pocket of 7BQY is shown with and without the amino acid residues (Figure 5). Docking scores were considered to be the binding affinities in kcal/mol unit, with more negative values demonstrating enhanced critical strength [29] (Figure 4).

**Figure 6:**
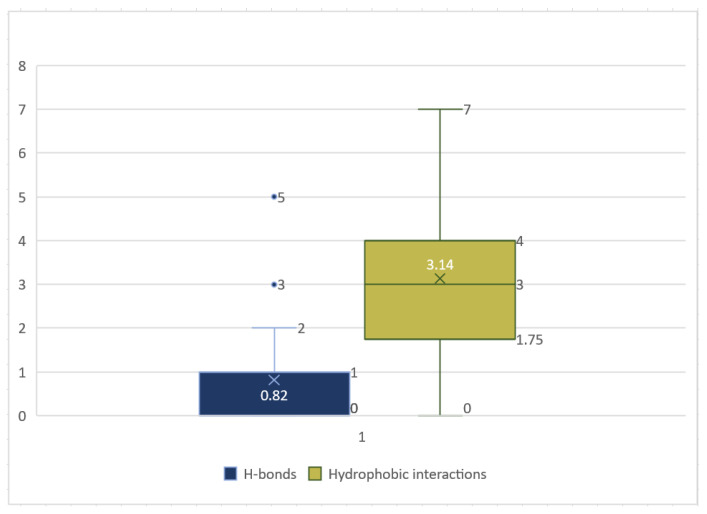
A box-whisker plot represents an excellent graphical image of the concentration of the data of the number of hydrogen and hydrophobic bonds among amino acid residues of the COVID-19 substrate binding site to the newly synthesized SB derivatives **5a**–**g**as a result of the docking analysis carried out, **S1**.

**Figure 7:**
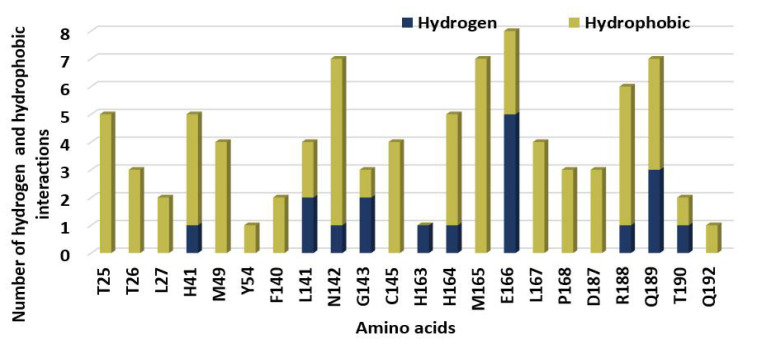
The number of hydrophobic interactions and H-bonds revealed upon docking of 7BQY protease against the SBs.

The identified hydrogen and hydrophobic interactions between the SBs and 7BQY amino acid residues of COVID-19formed due to docking are presented in Figure 6. Amino acid E166 showed the highest H-bonds and also the highest hydrogen and hydrophobic interactions in total. Y54 and Q192 were the least important amino acid residues. H163 only offered hydrogen bonds, whereas T25, T26, M49, Y54, F140, C145, M165, L167, P168, D187, and Q192 displayed only hydrophobic interactions (Figure 7).

A box-whisker plot displayed the concentration of the number of hydrogen and hydrophobic interactions between the amino acid residues of COVID-19 and the newly synthesized SBs. The number of hydrogen bonds varied between the seven compounds, docked with the COVID-19 M^pro^ pocket, with a median of 0.82. The highest number of H-bonds observed was 5 bonds for **5e**, and the lowest was 1 bond for **5b**, **5c**, and **5f**. The lowest H-bonds were 0, and the maximum value was 5 and 2 for the maximum before the upper fence. The hydrophobic interaction found between the SBs and M^pro^pocket ranged from 7 and 14 with average and median values of 3.14 and 3, respectively. The highest value was 14 for compound 5f with a maximum value before the fence of 7 (Figure 6 and Figure S1).

**Figure 8:**
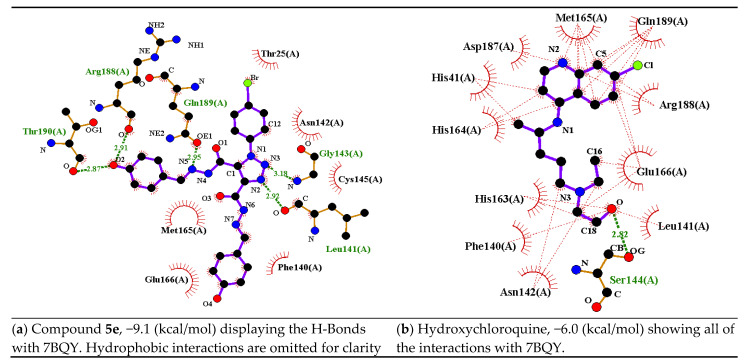
A schematic of 2D LIGPLOT drawings of **5e** and hydroxychloroquine against 7BQY protease using the 2D LIGPLOT representation of the protein–ligand interactions [44]; (**a**) a diagram showing the hydrogen bonds and hydrophobic residues of **5e** with 7BQY; (**b**) a diagram showing the hydrogen bonds and hydrophobic residue interactions of hydroxychloroquine with 7BQY protease. A key to the symbols is reported before [45]. The cutoff for the nonbonded interactions is 3.9 Å.

A comparison between compound **5e** and hydroxychloroquine in terms of the number of interactions (H-bonding: green dashed lines; hydrophobic: thin red dashed lines) and binding affinity values are presented in Figure 8 as a representative example. Both the ligands have halogen atoms and hydroxyl groups. Compound **5e** exhibited 5 H-bonds: Leu141 (2.92 Å), Gly143 (3.18 Å), Gln189 (2.95 Å), Arg188 (2.91 Å), and Thr190 (2.87 Å). It showed hydrophobic interactions with Phe140, Leu141, Cys145, Gly143, Asn142, Thr25, Gln189, Arg188, Thr190, Met165, and Glu166 (Figure 8a). Hydroxychloroquine showed only one H-bond with Ser144 (2.82 Å), whereas it showed many hydrophobic interactions with Asn142, Ser144, Leu141, Glu166, Arg188, Phe140, His163, His41, Asp187, Met165, andGln189 (Figure 8b). It could be concluded from this example that the better interaction, the lower the binding affinity [43].

**Figure 9:**
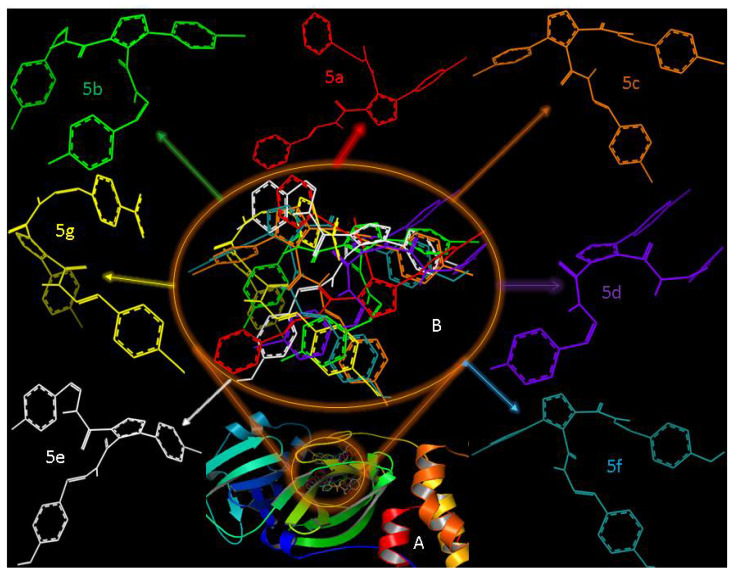
A: The display of the superposition of all the SBs, **5a**–**g**, docked against 7BQY; B: the display of the ligands without the 7BQY protease surrounded by the display of each ligand as they appeared in the ligand–protein binding cavity.

Comparative docking analysis was carried out to evaluate each ligand’s display, after docking, in light of different Y groups (Scheme 1, Figure 9). Surprisingly, although all ligands possessed the same 1,2,3-triazole motif, the ligands interacted differently against 7BQY due to, probably, the different Y group at the *para* position of each ligand (Figure 9). Ligands **5a**, **5c**, and **5f** are close in their apparent appearance, yet this different behavior of the ligands was not reflected in the binding affinities of the ligands against 7BQY (Figure 5). Ligand **5e** showed the highest binding affinity due to, probably, the two hydroxyl groups in the *para* position, creating much interaction in the protein cavity (Figure 8a). Hence, it may not be possible to anticipate the ligands’ relative binding affinities based on the display of the ligands in the protein cavity.

**Figure 10:**
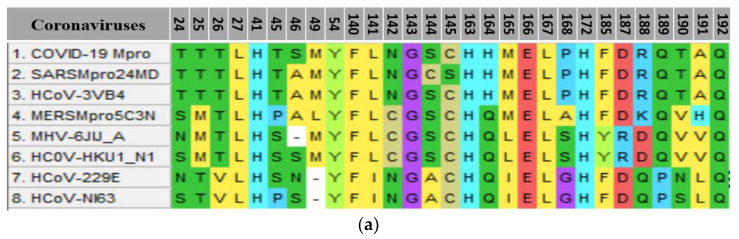

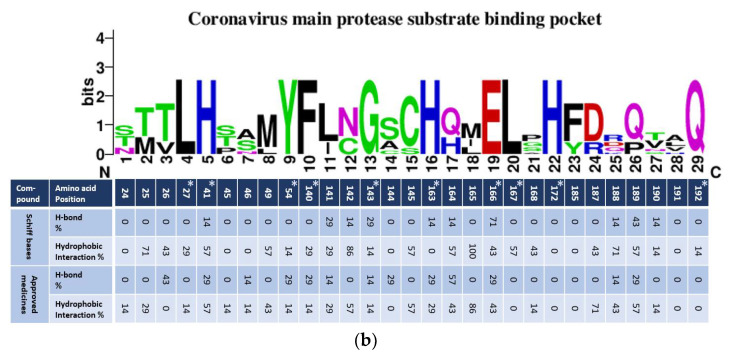
(**a**) Alignment of amino acid constructing coronavirus substrate-binding pockets in the M^pro^. (**b**) A WebLogo analysis of amino acid residues constructing the substrate-binding pocket consensus of coronaviruses’M^Pro^. The table runs parallelly with the amino acid consensus, showing the percentage calculated for H-bonds and hydrophobic interactions resulting from in silico studies using protein docking. Results were carried out for two groups of ligands, the newly synthesized SBs, and the approved medicines used to treat COVID-19.

A detailed comparative analysis was carried out to evaluate the interactions of the SBs, **5a**–**g**, the approved medicines, and **N3** with the coronaviruses. Generated alignment of the residues constructing the substrate-binding pockets of human coronaviruses 229E, OC43, NL63, HKU1, SARS, and MERS (Figure 10a) shared 10 conserved amino acids, Leu27, His41, Tyr54, Phe140, G143, His163, Glu166, Leu167, His172, and Gln192. The SBs and the approved medicines’ docking outcomes were calculated as percentages for each amino acid for both the groups against COVID-19 [41]. The COVID-19 binding pocket was comparable to other coronaviruses [46,47,48,49,50] (Figure 10a,b).

Binding pocket residue interactions with the Schiff bases **5a**–**g** were calculated. Results were presented in parallel with consensus amino acid sequences constructing substrate-binding pockets generated with WebLogo (Figure 10b). Docking results showed that most of the conserved amino acids among coronaviruses, including COVID-19, were involved in at least one interaction, except His172. Two polymorphic amino acids did not interact with either H-bond or hydrophobic at positions 185 and 191 with both ligands (Figure 10b). H-bonds were not observed with two conserved leucines at positions 27 and 167 with both the ligands. The other seven conserved amino acids showed varied H-bond percentage interactions with both the ligands (Figure 10). Four of them, His41, G143, His163, and Glu166, displayed H-bonds against most of the SBs (**5a**, **5c**, **5f**, and **5g**) with the highest calculating percentage being 71% for Glu166. However, two approved drugs (ribavirin and remdesivir) interacted with Glu166. The second-highest percentage was 29% of H-bonds for SBs **5d** and **5e** against the conserved amino acid at 143 (Figure 10b and Figure S1). Ligand **N3** was the only one that showed interaction with G143 (Figure S10). In general, the approved medicines generated six high numbers of H-bonds of the conserved amino acids out of 10, with the highest percentage being 29 (Figure 10b).

Higher percentages were calculated for hydrophobic interactions against the conserved amino acids for coronaviruses against the ligands. Equal percentages calculated, 57, 14, and 14 of hydrophobic interactions against the three conserved amino acids, His41, Tyr54, and G143, respectively. Hydrophobic interaction percentages against the other six conserved amino acids formed by the SBs against, Leu27, Phe140, His163, Glu166, Leu167, and Gln192 were 43, 29, 0, 43, 57, and 14, respectively. The approved medicines interacted hydrophobically against four of the conserved amino acids, Leu27, Phe140, His163, and Glu166, with 14, 14, 29, and 57 percentages, respectively, Figure 10b.

Percentages calculated for the hydrogen and hydrophobic interactions against the polymorphic amino acids constructed the substrate pocket for coronaviruses. Position 165, amino acid, showed the highest calculated percentage (100%) for all ligands. This amino acid at 165 was in SARS, 3VB4, and MERS, but not in other coronaviruses. The second-highest percentage calculated was 86% against an amino acid at position N142. All ligands formed hydrophobic interactions with the amino acid at N142, except **5a** (Figure 10b). This amino acid was found in SARS, 3VB4, 229E, and NL63 coronaviruses (Figure 10a). The third-highest percentage calculated for the SBs was 71%, with the amino acids at Thr25 and R188. The two SBs that did not interact with Thr25 were in SARS, 3VB4, 229E, and NL63. The approved medicineshydroxychloroquine, favipiravir, and remdesivir, also interacted with R188 hydrophobically (Figure 10a,b and Figure S2). The remaining polymorphic amino acids showed hydrophobic interactions against the ligands ranged between 0 to 57% (Figure 10b).

The dihedral angles (φ, C14, C10, C9, and C13*)*, the number of flexible bonds, and binding affinities were calculated for **5a**–**g** and are given in Table 3 for a correlation assessment, if any. Flexible bonds were automatically identified by AutoDock [51]. The dihedral angles werecalculatedusingPyMOL [42] for the molecules before and after the docking process, showing negligible differences. Unexpectedly, the results in Table 3, suggest no visible direct correlations among the three physical properties. However, it was found that the number of ligand conformational degrees of freedom was the main aspect of the active sites in docking and the internal dihedral angles [52]. Yet, neither dihedral angles nor binding affinities were related to the number of flexible torsions in the ligand in this study.

A recent study by A-T. Ton and co-workers identified the highest 1000 potential ligands for SARS-CoV-2 M^pro^ by applying the deep docking method in conjunction with the glide method to around 1.3 billion compounds obtained from the Zinc-15 database [53,54]. The maximum potential docked molecules displayed docking scores ranging from −11.32 to −9.00 kcal/mol, comparable to our results [55]. Based on these docking results, it can be anticipated that the SBs **5a**–**g** could be sound candidates against COVID-19 for possible medicinal drugs.

#### In Silico Cytotoxic Effect on Human Cancer Cell Lines

PASS results for the SBs **5a**–**g** are presented in Table 4 and Table 5. The predicted bioactivities were varied in their functionality. The first six predicted activities were presented in these two tables. The first activity predicted for **5a**–**g** was the mitochondrial enzyme HMGCS2 (3-hydroxy-3-methylglutaryl-CoA synthase, 2) expression enhancer in the range 0.85–0.89. The HMGCS2 protein contributes to regulating intestinal cell differentiation [56]. Another two bioactivities related to tuberculosis (TB); antimycobacterial (drugs used to treat mycobacterium genus include TB) and antituberculosis drugs were in the range of 0.78–0.85 and 0.68–0.79, respectively (Table 4). The PfA-M1 aminopeptidase inhibitor (antimalarial) predicted bioactivity ranged from 0.66 to 0.73 (Table 4). Another activity was age-related macular degeneration (AMD) and it ranged from 0.59 to 0.64. The last two bioactivities predicated by PASS were inhibitors, orexin receptor 1 and Mcl-1, which were in the ranges of 4.45–0.57 and 0.48–0.60, respectively (Table 4). PASS predication bioactivity on nontumor cells was also varied (Table 5). Ligands, except **5f**, had activity as the skin condition neutrophilic dermatosis ranged between 0.67–0.30. The compounds **5a**, **5b,** and **5d** had bioactivity as nail discoloration in Pa > 0.35, 0.35, and 0.3, respectively. Bioactivities predicated for **5c** were 0.31 and 0.34 as adrenal cortex hypoplasia and multiple organ failure, respectively. The acneiform eruption and gastrointestinal hemorrhage bioactivities were predicated only for **5d** (Pa > 0.31) and **5e** (Pa > 0.38), respectively. No bioactivity was predicted by PASS for compound 5f in nontumor cells (Table 5). It was shownthat1,2,3-triazole derivatives displayed a wide range of biological activities through diverse mechanisms. Therefore, they can be anticancer, antiviral, antitubercular, anti-inflammatory, antibacterial, antileishmanial and antitrypanosomal, and antimicrobial, among others [33]. This study’s products also displayed a wide range of biological mechanisms such as anticancer, antibacterial, and others (Table 4 and Table 5). The carboxyamidotriazole (CAI) standard as an anticancer drug [34] was used to compare in PASS. Predicted bioactivities were exhibitedas anticancer (Pa > 0.75), antiprotozoal (Pa > 0.74), antipsoriatic (Pa > 0.66), angiogenesis inhibition (Pa > 0.57), and antineoplastic (Pa > 0.56). 

### 2.3. Quantitative Structure-Activity Relationships (QSAR)

The QSAR investigation was achieved by the calculation of some thermodynamic molecular parameters such as hydrophobicity (LogP), volume, polar surface area, ovality, dipole moment (DM), and highest occupied molecular orbital (HOMO) and lowest unoccupied molecular orbital (LUMO) energy levels (Table 3). Recently, biological activities such as antifungal, anticancer, antimicrobial, and cytotoxic activities and new-drug-design fields have been investigated in terms of frontier orbitals levels and their energy differences. It is well known that HOMO and LUMO as frontier molecular orbitals are vital parameters to give qualitative information about the electronic excitation of such prepared compounds (**5a**–**g**) 14–18. Moreover, the energy gap between frontier molecular orbitals and their levels plays an essential role in predicting the degree of binding and energy required for the compound to bind to the proteins. Thus, calculation of such parameters could give a vision about the degree and the type of hydrophilic interactions and H-bonding of the compounds under investigation and the desired receptor in nonbonding intermolecular interactions. The energy level of HOMOs is a prediction of the capability of the compound for electronic donation to the receptors 18–20. However, the energy of LUMOs is a qualitative parameter for their electronic acceptance from the receptors 18–20.

On the other hand, several chemical descriptors could also be calculated from the FMOs energy levels, such as the electronegativity (χ) and the Lewis acidity ability of the compounds. The global hardness (η) is the magnitude of the charge transfer hindrance. The electrophilicity (*ω*) is the amount of energy of electronic transition and could be estimated from the electronegativity and chemical hardness values.

**Figure 11:**
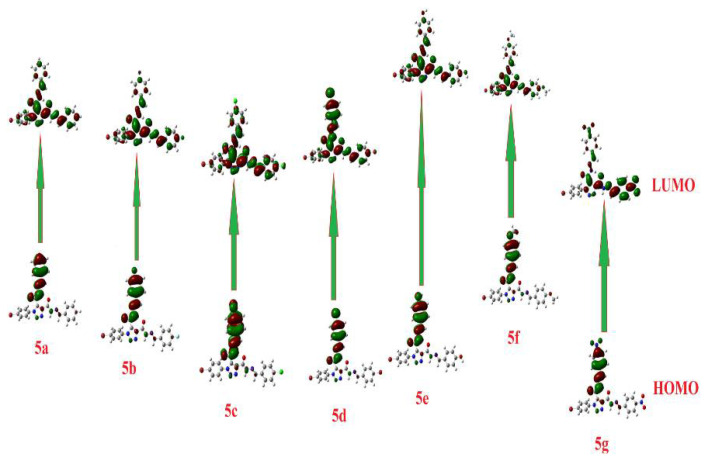
The calculated ground state isodensity surface plots for FMOs for the prepared compounds **5a**–**g**.

The calculated ground state isodensity surface plots for FMOs for the prepared compounds **5a**–**g** are illustrated in Figure 11. The energy levels of HOMOs were in the range of −5.67 to −6.82 eV; however, those of the LUMOs were −2.23 eV to −3.27 eV. Since compound **5g** showed the least laying HOMO and LUMO, −6.82 and −3.27 eV, respectively, compound **5f** showed the highest HOMO and LUMO, −5.67 and −2.23 eV, respectively. This may explain why **5g** and **5f** have low binding energies, −8.0 and −8.5 kcal/mole, respectively. On the other hand, the energy level of **5f** of the high laying LUMO can receive electrons from the receptor.

The energy gap between the compounds’ FMOs depends on the electronic nature of the attached groups and the degree of conjugation of the unsaturated part. The FMOs energy gaps were in the range 3.44–3.71 eV. It is evident that there is no significant effect of the electronic nature of the attached group where there is no conjugation of the aromatic ring carrying the subsistent and the central phenyltriazole. From Table 2, it is clear that compound **5g** showed the highest basicity score, χ = 5.05, and could be another illustration for its expected high inhibitory activity.

On the other hand, the estimation of lipophilicity is vital in predicting whether drugs will pass through several membranes of the biological organs, and it reveals the cytotoxicity of the compounds. Moreover, it shows the fluidity of the molecule in lipophilic parts. The calculated log P demonstrated that compound **5e** showed the least at 3.87; however, **5d** was at 6.36, and such variation between the prepared compounds suggest a preferable one rather than the other for enzyme inhibition. Additionally, the magnitude of the H-bonding donation or acceptance of the drug candidates could be good parameters to illustrate the extent of binding of them and the protein and the type of these interactions. Compounds **5g** and **5e** showed the highest, with respect to other compounds, of H-bonding acceptance and donations, respectively.

## 3. Experimental 

### 3.1. Synthesis

All reagents and solvents used were of the highest quality of analytical reagent grade and were used without further purification. Fine chemicals and solvents were purchased from BDH Chemicals Ltd. (Poole, UK) and Sigma-Aldrich (St. Louis, Missouri, USA). Melting points were measured on a Stuart Scientific SMP1 (Stuart, UK) and were uncorrected. TLC was performed on UV fluorescent Silica gel Merck 60 F254 plates, and the spots were visualized using a UV lamp (254 nm). A Perkin-Elmer, USA, 1430 series FT-IR spectrometer was used to identify functional groups in the range 400–4000 cm^−1^. The NMR spectra were run with a 400 MHz Bruker spectrometer (Bruker, Switzerland) with TMS as an internal reference. Elemental analyses were performed using a GmbH-Vario EL III Elementar Analyzer (GmbH, Germany).

**Synthesis and characterization of dimethyl 1-(4-bromophenyl)-1*H*-1,2,3-triazole-4,5-dicarboxylate (3)**

A mixture of dimethylacetylene dicarboxylate (**1**) (15 mmol, 2.13 gm) and 1-azido-4-bromobenzene (**2**) (20 mmol, 3.96 gm) were heated to 80–90 °C for 3 min. After cooling, ether (20 mL) was added, and the precipitate formed was collected via filtration and purified viacrystallization with ethanol. Yield: 92%; mp 89–90 °C. IR (KBr, υ): 3070 (C–H_ar_), 2965 (C–H_al_), 1725 cm^−1^ (C=O). ^1^H-NMR (400 MHz, DMSO-*d*_6_): δ_H_: 3.88 (3H, s, OC***H***_3_), 3.94 (3H, s, OC***H*_3_**), 7.65 (2H, d, *J* = 8 Hz, Ar***H***), 7.88 (2H, d, *J* = 8 Hz, Ar***H***). ^13^C-NMR (100 MHz, DMSO-*d*_6_): δ_C_: 52.77, 54.00 (2 ×O***C***H_3_); 124.03, 126.83, 131.73, 132.77, 134.34, 138.48 (Ar***C***); 158.50, 159.79 (2 ×***C***=O). Anal. Calcd. for C_12_H_10_BrN_3_O_4_: C, 42.37; H, 2.96; N, 12.35. Found: C, 42.62; H, 2.87; N, 12.24.

**Synthesis and characterization of 1-(4-bromophenyl)-1*H*-1,2,3-triazole-4,5-dicarbohydrazide (4)**

A solution of ester **3** (20 mmol, 6.8 gm) and hydrazine hydrate (40 mmol, 1.2 gm) in ethanol (30 mL) was refluxed for 4 h until the consumption of the starting materials (as monitored using TLC). After cooling to room temperature, ethanol was removed under reduced pressure and the resulting solid was recrystallized from ethanol. Yield: 90%; mp > 300 °C. IR (KBr, υ): 3225–3385 (NH, NH_2_), 3085 (C–H_ar_), 2945 (C–H_al_), 1690 cm^−1^ (C=O). ^1^H-NMR (400 MHz, DMSO-*d*_6_): δ_H_: 4.73 (4H, brs, 2 × N***H*_2_**), 7.54 (2H, d, *J* = 12 Hz, Ar***H***), 7.80 (2H, d, *J* = 12 Hz, Ar***H***), 10.95 (2H, brs, 2 × N***H***). ^13^C-NMR (100 MHz, DMSO-*d*_6_): δ_C_: 123.05, 127.23, 132.08, 135.74, 138.50 (Ar***C***); 155.45, 158.50 (2 ×***C***=O). Anal. Calcd. for C_10_H_10_BrN_7_O_2_: C, 35.31; H, 2.96; N, 28.83. Found: C, 35.54; H, 2.84; N, 28.97.

**General method for the synthesis of Shiff bases 5a–g.**

A mixture of bis-hydrazide **4** (1 mmol) and the appropriate aromatic aldehydes (2 mmol) in ethanol (20 mL) with few drops of HCl was refluxed 2–4 h. After cooling, the product obtained was collected and recrystallized from ethanol/DMF.

***Characterization of N’^4^,N’^5^-dibenzylidene-1-(4-bromophenyl)-1H-1,2,3-triazole-4,5-dicarbohydrazide* (5a)**

90% Yield of compound **5a**, mp > 300 °C. IR (KBr, υ): 3340 (NH), 3070 (C–H_ar_), 2920 (C–H_al_), 1695 cm^−1^ (C=O). ^1^H-NMR (400 MHz, DMSO-*d*_6_): δ_H_: 7.32–7.34 (2H, m, Ar***H***), 7.43–7.48 (5H, m, Ar***H***), 7.59–7.68 (3H, m, Ar***H***), 7.74–7.77 (2H, m, Ar***H***), 7.85 (2H, t, *J* = 8 Hz, Ar***H***), 8.04 (0.50H, s, ***H***C=N), 8.30 (0.50H, s, ***H***C=N), 8.57 (0.50H, s, ***H***C=N), 8.68 (0.50H, s, ***H***C=N), 12.04 (0.15H, s, N***H***), 12.43 (0.65H, s, N***H***), 12.64 (1.20H, brs, N***H***). ^13^C-NMR (100 MHz, DMSO-*d*_6_): δ_C_: 123.54, 123.59, 125.70, 126.85, 126.90, 127.13, 127.28, 127.45, 128.76, 128.80, 128.87, 130.21, 130.30, 130.44, 130.69, 132.59, 133.01, 133.28, 133.51, 133.65, 134.03, 134.09, 134.29, 134.58, 135.07, 139.23, 140.12, 146.22, 148.86, 149.60, 149.77 (***C_arom_***), 153.17, 155.33, 156.07, 160.04 (***C***=O, ***C***=N). Anal. Calcd. for C_24_H_18_BrN_7_O_2_: C, 55.83; H, 3.51; N, 18.99. Found: C, 55.68; H, 3.60; N, 18.88. 

***Characterization of 1-(4-bromophenyl)-N’^4^,N’^5^-bis(4-fluorobenzylidene)-1H-1,2,3-triazole-4,5-dicarbohydrazide* (5b)**

89% Yield of compound **5b**, mp 283–284 °C. IR (KBr, υ): 3320 (NH), 3050 (C–H_ar_), 2960(C-H_al_), 1685 cm^−1^ (C=O). ^1^H-NMR (400 MHz, DMSO-*d*_6_): δ_H_: 7.41–7.84 (12H, m, Ar***H***), 8.00 (0.50H, s, ***H***C=N), 8.28 (0.50H, s, ***H***C=N), 8.52–8.72 (1H, 3 s, ***H***C=N), 12.12 (0.15H, s, N***H***), 12.49 (0.65H, s, N***H***), 12.75 (1.20H, brs, N***H***). ^13^C-NMR (100 MHz, DMSO-*d*_6_): δ_C_: 116.15, 123.19, 127.42, 130.38, 130.70, 131.91, 134.45, 160.41, 165.11 (***C_arom_***, ***C***=O, ***C***=N). ^19^F-NMR: δ_F_: −108.54 to −108.46.Anal. Calcd. for C_24_H_16_BrF_2_N_7_O_2_: C, 52.19; H, 2.92; N, 17.75. Found: C, 52.38; H, 2.82; N, 17.90. 

***Characterization of 1-(4-bromophenyl)-N’^4^,N’^5^-bis(4-chlorobenzylidene)-1H-1,2,3-triazole-4,5-dicarbohydrazide* (5c)**

87% Yield of compound **5c**, mp 254–255 °C. IR (KBr, υ): 3350 (NH), 3040 (C–H_ar_), 2900 (C–H_al_), 1690 cm^−1^ (C=O). ^1^H-NMR (400 MHz, DMSO-*d*_6_): δ_H_: 7.41–8.01 (12H, m, Ar***H***), 8.28 (0.50H, s, ***H***C=N), 8.53 (0.50H, s, ***H***C=N), 8.64 (0.50H, s, ***H***C=N), 8.71 (0.50H, s, ***H***C=N), 12.08 (0.15H, s, N***H***), 12.47 (0.65H, s, N***H***), 12.74 (1.20H, brs, N***H***). ^13^C-NMR (100 MHz, DMSO-*d*_6_): δ_C_: 123.56, 125.75, 126.81, 128.99, 129.99, 132.60, 132.99, 134.75, 139.21, 145.02, 147.55, 148.36 (***C_arom_***), 155.38, 156.12, 160.08, 160.60 (***C***=O, ***C***=N).Anal. Calcd. for C_24_H_16_BrCl_2_N_7_O_2_: C, 49.25; H, 2.76; N, 16.75. Found: C, 49.45; H, 2.84; N, 16.87. 

***Characterization of N’^4^,N’^5^-bis(4-bromobenzylidene)-1-(4-bromophenyl)-1H-1,2,3-triazole-4,5-dicarbohydrazide* (5d)**

87% Yield of compound **5d**, mp 275–276 °C. IR (KBr, υ): 3310 (NH), 3080 (C–H_ar_), 2900 (C–H_al_), 1690 cm^−1^ (C=O). ^1^H-NMR (400 MHz, DMSO-*d*_6_): δ_H_: 7.36 (1H, d, *J* = 8 Hz, Ar***H***), 7.53 (1H, d, *J* = 8 Hz, Ar***H***), 7.58–7.73 (8H, m, Ar***H***), 7.81–7.86 (2H, m, Ar***H***), 8.00 (0.50H, s, ***H***C=N), 8.27 (0.50H, s, ***H***C=N), 8.51 (0.50H, s, ***H***C=N), 8.63 (0.50H, s, ***H***C=N), 12.09 (0.15H, s, N***H***), 12.47 (0.65H, s, N***H***), 12.74 (1.20H, brs, N***H***). ^13^C-NMR (100 MHz, DMSO-*d*_6_): δ_C_: 123.48, 123.57, 123.60, 123.71, 124.03, 125.76, 126.81, 128.74, 128.96, 129.09, 129.28, 130.17,131.77, 131.82, 131.90, 132.51, 132.60, 132.82, 132.99, 133.31, 133.34, 134.12, 134.55, 139.21, 140.15, 145.16, 147.66, 148.46 (***C_arom_***), 153.28, 155.39, 156.08, 159.99, 160.72 (***C***=O, ***C***=N).Anal. Calcd. for C_24_H_16_Br_3_N_7_O_2_: C, 42.76; H, 2.39; N, 14.54. Found: C, 42.58; H, 2.28; N, 14.67. 

***Characterization of 1-(4-bromophenyl)-N’^4^,N’^5^-bis(4-hydroxybenzylidene)-1H-1,2,3-triazole-4,5-dicarbohydrazide* (5e)**

88% Yield of compound **5e**, mp > 300 °C. IR (KBr, υ): 3280–3390 (NH, OH), 3080 (C–H_ar_), 2890 (C–H_al_), 1685 cm^−1^ (C=O). ^1^H-NMR (400 MHz, DMSO-*d*_6_): δ_H_: 6.73 (1H, d, *J* = 8 Hz, Ar***H***), 6.82–6.88 (3H, m, Ar***H***), 7.23–7.26 (1H, m, Ar***H***), 7.53 (1H, d, *J* = 8 Hz, Ar***H***), 7.57–7.64 (4H, m, Ar***H***), 7.81–7.86 (2H, m, Ar***H***), 7.91 (0.50H, s, ***H***C=N), 8.17 (0.50H, s, ***H***C=N), 8.45 (0.50H, s, *H*C=N), 8.55 (0.50H, s, ***H***C=N), 9.98 (1H, brs, O***H***), 10.03 (1H, brs, O***H***), 12.17 (0.50H, s, N***H***), 12.41 (1H, brs, N***H***), 12.80 (0.50H, brs, N***H***). ^13^C-NMR (100 MHz, DMSO-*d*_6_): δ_C_: 115.63, 115.68, 115.76, 123.43, 123.47, 124.31, 124.47, 124.99, 125.10, 125.64, 126.91, 128.71, 128.93, 129.13, 129.32, 132.49, 132.96, 133.55, 134.31, 134.67, 135.24, 139.13, 140.17, 146.34, 149.06, 149.72(***C_arom_***), 150.10, 152.71, 155.06, 155.87, 159.51, 159.54, 159.68, 158.73, 159.94 (***C***=O, ***C***=N).Anal. Calcd. for C_24_H_16_Br_3_N_7_O_2_: C, 42.76; H, 2.39; N, 14.54. Found: C, 42.58; H, 2.28; N, 14.67. 

***Characterization of 1-(4-bromophenyl)-N’^4^,N’^5^-bis(4-methoxybenzylidene)-1H-1,2,3-triazole-4,5-dicarbohydrazide* (5f)**

88% Yield of compound **5f**, mp 286–287 °C. IR (KBr, υ): 3330 (NH), 3040 (C–H_ar_), 2970 (C–H_al_), 1700 cm^−1^ (C=O). ^1^H-NMR (400 MHz, DMSO-*d*_6_): δ_H_: 3.71 (1.50H, s, OC***H***_3_), 3.76 (0.50H, s, OC***H***_3_), 3.79 (1.25H, s, OC***H***_3_), 3.81 (2.75H, s, OC***H***_3_), 6.89 (1H, d, *J* = 8 Hz, Ar***H***), 6.99–7.05 (3H, m, Ar***H***), 7.36 (1H, d, *J* = 8 Hz, Ar***H***), 7.57–7.64 (3H, m, Ar***H***), 7.70 (2H, d, *J* = 8 Hz, Ar***H***), 7.83–7.86 (2H, m, Ar***H***), 7.96 (0.50H, s, ***H***C=N), 8.23 (0.50H, s, ***H***C=N), 8.49 (0.50H, s, ***H***C=N), 8.59 (0.50H, s, ***H***C=N), 12.24 (0.80H, s, N***H***), 12.43 (1.20H, brs, N***H***). ^13^C-NMR (100 MHz, DMSO-*d*_6_): δ_C_: 55.19 (***C***H_3_O), 55.26 (***C***H_3_O), 55.30 (***C***H_3_O), 114.24, 114.30, 114.38, 123.48, 125.66, 126.09, 126.56, 126.66, 126.90, 128.54, 128.75, 128.93, 129.12, 132.51, 132.97, 139.14, 145.97, 148.64, 149.36, 149.63 (***C_arom_***), 152.93, 155.14, 155.95, 159.78, 160.89, 160.90, 161.09, 161.25 (***C***=O, ***C***=N).Anal. Calcd. for C_26_H_22_BrN_7_O_4_: C, 54.18; H, 3.85; N, 17.01. Found: C, 54.34; H, 3.83; N, 17.10. 

***Characterization of 1-(4-bromophenyl)-N’^4^,N’^5^-bis(4-nitrobenzylidene)-1H-1,2,3-triazole-4,5-dicarbohydrazide* (5g)**

87% Yield of compound **5g**, mp 271–272 °C. IR (KBr, υ): 3300 (NH), 3050 (C–H_ar_), 2930 (C–H_al_), 1680 cm^−1^ (C=O). ^1^H-NMR (400 MHz, DMSO-*d*_6_): δ_H_: 7.80–7.87 (3H, m, Ar***H***), 8.02–8.09 (3H, m, Ar***H***), 8.17–8.22 (2H, m, Ar***H***), 8.32–8.34 (1.5H, m, Ar***H***), 8.45–8.52 (3H, m, Ar***H***, ***H***C=N), 8.60 (0.50H, s, ***H***C=N), 8.82 (0.50H, s, ***H***C=N), 8.95 (0.50H, s, ***H***C=N), 12.56 (0.15H, s, N***H***), 12.92 (0.65H, s, N***H***), 13.15 (1.20H, brs, N***H***). ^13^C-NMR (100 MHz, DMSO-*d*_6_): δ_C_: 123.66, 123.92, 124.03, 124.12, 125.88, 126.77, 127.89, 128.02, 128.43, 132.67, 133.00, 133.71, 133.99, 134.50, 134.88, 138.40, 139.40, 139.77, 140.26, 144.15, 146.47, 147.23, 147.37, 147.90, 148.04, 148.20 (***C_arom_***), 153.69, 155.65, 156.24, 160.23 (***C***=O, ***C***=N).Anal.Calcd. for C_24_H_16_BrN_9_O_6_: C, 47.54; H, 2.66; N, 20.79. Found: C, 47.38; H, 2.74; N, 20.65.

### 3.2. Docking in Silico Studies

Docking studies of the Schiff bases as ligands were performed using the Autodock Vina wizard in PyRx 0.8 (available freely from https://sourceforge.net/projects/pyrx/, accessed on: 16 September 2020) [35] against 7BQY (PDB, resolution**:** 2.16 Å). The program’s settings included a grid box (25, 25, 25 Å) centered at 6.01698333056, −1.96966661824, 21.7763995317; and exhaustiveness of 8; the number of active torsions was set to 7–10 bonds for the flexible docking ligand mode. A boxplot was used to illustrate the variation of H-bonds and hydrophobic interactions of the seven newly synthesized SBs with COVID-19 substrate-binding pockets. Alignment of the 29 amino acids was carried out using MEGA X [57].To study the amino acid residue variations involved in the M^pro^ substrate-binding pockets conformational of different coronaviruses, WebLogo was used [58,59]. Firstly, 29 amino acids were numbered as follows; Thr24, Thr25, Thr26, Leu27, His41, T45, S49, Met49, Tyr54, Phe 140, Leu141, N142, G143, Ser144, Cys145, His163, His164, Met165, E166, Leu167, Pro168, His172, Phe185, Asp187, Arg188, Glu189, Thr190, Ala191, and Glu192 as they were identified in COVID-19 [41,60]. Secondly, the same residue numbers were collected from different human coronaviruses, 229E, -OC43, -NL63, -HKU1, SARS, and MERS. M^pro^ amino acid sequences for HCoV-229E, HCoV-OC43, HCoV-NL63, HCoV-HKU1, MERS-CoV, SARS-CoV, and NCBI were found online at https://www.ncbi.nlm.nih.gov/ (accessed on: 16 September 2020). Thirdly, amino acid constructing substrate-binding pockets of coronaviruses, including COVID-19, were aligned using WebLogo, which is a web-based application (available online: https://weblogo.berkeley.edu/, accessed on: 16 September 2020).

### 3.3. In Silico Cytotoxic Effect on Human Cancer Cell Lines

Activity spectra for the synthesized SBs **5a**–**g** prediction was carried out in silicon using the Predication Activity Spectra for Substances (PASS) (available online: https://www.way2drug.com/, accessed on: 16 September 2020), which is software used to predict drug-like substances’ biological activity. The spectrum produced by PASS for a substance can be labeled as probable activity (Pa) and probable inactivity (Pi).

## 4. Conclusions

A novel series of Schiff bases of 1,2,3-triazole motifs were synthesized and modeled. The spectroscopic data of the synthesized compounds showed the presence of E/Z geometrical isomers and the cis/trans conformers. The DFT results of assumed geometrical isomers ***trans-trans, syn-syn***, and ***trans-syn*** based on the orientation of the two amidic (C=O and N–H) groups to each other of both arms of the bis-acid hydrazones revealed the stability of ***trans***-***trans*** by 9.26 and 5.56 kcal/mol with respect to the other isomers, and it was attributed to the formation of stronger intramolecular H-bonds. Our novel Schiff base derivatives have been presented as possible inhibitors of COVID-19 using a molecular docking method. The binding affinities of the SBs in this study against 7BQY were in the range of −7.3 to −9.1 kcal/mol. Remarkably, the result showed that the studied SBs had a comparable binding affinity for the main protease (PDB code 7BQY) using the same parameters to those of approved medicines, From the obtained molecular docking and QSAR results, these SBs could be considered for further investigation in the context of possible therapeutic agents for COVID-19.

## Figures and Tables

**Table 1:** Thermal parameters (hartree/particle) of geometrical isomers of the chloro derivative 5c.

Parameter	5c *Cis-Cis*	5c *Cis-Trans*	5c *Trans-Trans*
**E_corr_**	0.366677	0.367067	0.367365
**ZPVE**	−4952.400554	−4952.406195	−4952.414964
**E*_tot_***	−4952.368837	−4952.374717	−4952.383537
**H**	−4952.367893	−4952.373773	−4952.382593
**G**	−4952.472308	−4952.477581	−4952.486093
**ΔE in kcal/mol**	9.26	5.56	0.00

**Table 2:** Predicted H-bond length calculated by DFT at B3LYP 6-311G (d,p) basis set for **5a**–**g**.

Parameter	5a *Trans-Trans*	5b *Trans-Trans*	5c *Trans-Trans*	5d *Trans-Trans*	5e *Trans-Trans*	5f *Trans-Trans*	5g *Trans-Trans*
**H-bond length (Ǻ)**	1.73097	1.72869	1.72583	1.72985	1.72905	1.72551	1.72658

**Table 3:** Dihedral angles, number of flexible bonds, and binding affinities are presented here for comparison for the SBs **5a**–**g**.

**Comp. No.**	**Dihedral** **Angles, ϕ**	**No. of Flexible Bonds**	**Binding Affinities kcal/mol**	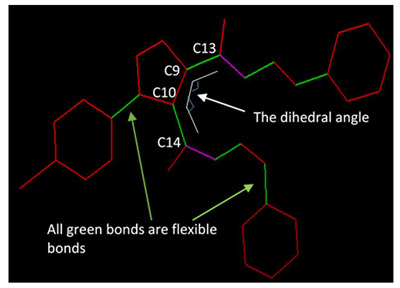 **5a** is a representative example to show the dihedral angles. Bonds in green are flexible bonds.
**5a**	2.4	7	−8.7	
**5b**	0.3	8	−8.5	
**5c**	7.0	7	−7.3	
**5d**	4.1	7	−7.6	
**5e**	7.2	7	−9.1	
**5f**	5.7	10	−8.0	
**5g**	6.3	10	−8.5	

**Table 4:** The predicted activities of the SBs **5a–g** on tumor cell lines using PASS at Pa > Pi.

Biological Activities on Tumor Cell Line.	5a		5b		5c		5d		5e		5f		5g	
	Pa	Pi	Pa	Pi	Pa	Pi	Pa	Pi	Pa	Pi	Pa	Pi	Pa	Pi
**HMGCS2 expression enhancer**	0.89	0.003	0.888	0.003	0.88	0.003	0.892	0.003	0.864	0.003	0.856	0.004	0.892	0.003
**Antimycobacterial**	0.79	0.004	0.787	0.004	0.783	0.004	0.789	0.004	0.798	0.004	0.791	0.004	0.859	0.003
**PfA-M1 aminopeptidase inhibitor**	0.73	0.003	0.729	0.003	0.71	0.003	0.736	0.003	0.692	0.004	0.666	0.004	0.721	0.003
**Antituberculosic**	0.7	0.004	0.703	0.004	0.681	0.004	0.702	0.004	0.725	0.004	0.682	0.004	0.799	0.003
**Age-related macular degeneration treatment**	0.61	0.003	0.605	0.003	0.611	0.003	0.608	0.003	0.593	0.004	0.646	0.003	0.633	0.003
**Orexin receptor 1 antagonist**	0.54	0.003	0.54	0.003	0.503	0.004	0.532	0.004	0	0	0.57	0.003	0.457	0.004
**Mcl-1 antagonist**	0.54	0.007	0.541	0.007	0.484	0.009	0.558	0.006	0.499	0.008	0.465	0.009	0.602	0.005

**Table 5:** The predicted activities of the SBs, **5a**–**g** on non-tumor cell lines using PASS at Pa > Pi.

Biological Activities on Non-Tumor Cell Line.	5a		5b		5c		5d		5e		5f		5g	
	Pa	Pi	Pa	Pi	Pa	Pi	Pa	Pi	Pa	Pi	Pa	Pi	Pa	Pi
**Neutrophilic dermatosis (Sweet’s syndrome)**	0.564	0.101	0.564	0.101	0.676	0.06	0.575	0.097	0.306	0.265	0	0	0.559	0.103
**Nail discoloration**	0.355	0.211	0.355	0.211	0	0	0.314	0.105	0	0	0	0	0	0
**Adrenal cortex hypoplasia**	0	0	0	0	0.316	0.129	0	0	0	0	0	0	0	0
**Multiple organ failure**	0	0	0	0	0.342	0.194	0	0	0	0	0	0	0	0
**Acneiform eruption**	0	0	0	0	0	0	0.314	0.105	0	0	0	0	0	0
**Gastrointestinal hemorrhage**	0	0	0	0	0	0	0	0	0.385	0.16	0	0	0	0

## Data Availability

Not applicable.

## Supplementary Materials

The following are available online at https://www.mdpi.com/article/10.3390/vaccines9091012/s1, Figure S1: ^1^H NMR of Compound **5a**; Figure S2: ^13^C NMR of Compound **5a**; Figure S3: ^1^H NMR of Compound **5b**; Figure S4: ^13^C NMR of Compound **5b**; Figure S5: ^19^F NMR of Compound **5b**; Figure S6: ^1^H NMR of Compound **5e**; Figure S7: ^13^C NMR of Compound **5e**; Figure S8: ^1^H NMR of Compound **5f**; Figure S9: ^13^C NMR of Compound **5f**; Figure S10: Ligand N3 was the only one that showed interaction with G143.

## References

[1] Narasimharao K. (2016). Design, Spectroscopic Characterization, Electrical Conductivity and Molecular Modelling Studies of Biologically Puissant Co(II) and Ni(II) Complexes of N,N’-bis(furan-2-ylmethyl)benzene-1,2- dicarboxamide. Int. J. Electrochem. Sci..

[2] Sharma O.P., Bhat T.K. (2009). DPPH antioxidant assay revisited. Food Chem..

[3] Florindo H.F., Kleiner R., Vaskovich-Koubi D., Acúrcio R.C., Carreira B., Yeini E., Tiram G., Liubomirski Y., Satchi-Fainaro R. (2020). Immune-mediated approaches against COVID-19. Nat. Nanotechnol..

[4] Asai A., Konno M., Ozaki M., Otsuka C., Vecchione A., Arai T., Kitagawa T., Ofusa K., Yabumoto M., Hirotsu T. (2020). COVID-19 drug discovery using intensive approaches. Int. J. Mol. Sci..

[5] World Health Organization (2020). CoVID-19 Strategy Up Date.

[6] Chaturvedi D., Kamboj M. (2016). Role of Schiff Base in Drug Discovery Research. Chem. Sci. J..

[7] Schiff H. (1864). Mittheilungenaus dem Universitätslaboratorium in Pisa: Eine neueReiheorganischerBasen. Justus Liebigs Ann. Chem..

[8] Bayrak H., Demirbas A., Karaoglu S.A., Demirbas N. (2009). Synthesis of some new 1,2,4-triazoles, their Mannich and Schiff bases and evaluation of their antimicrobial activities. Eur. J. Med. Chem..

[9] El-Naggar M., Abd El-All A.S., El-Naem S.I.A., Abdalla M.M., Rashdan H.R.M. (2020). New Potent 5α- Reductase and Aromatase Inhibitors Derived from 1,2,3-Triazole Derivative. Molecules.

[10] Kandile N.G., Mohamed M.I., Ismaeel H.M. (2017). Synthesis of new Schiff bases bearing 1,2,4-triazole, thiazolidine and chloroazetidine moieties and their pharmacological evaluation. J. Enzyme Inhib. Med. Chem..

[11] Khalaj A., Rastegi H.R., Jorjani M. (1998). Synthesis and muscle relaxant activity of two analogues of dantrolene sodium in mice. Pharm. Pharmacol. Commun..

[12] Sidorov N.G., Kravchenko A.D., Poddubikov A.V., Arzumanian V.G. (2019). Synthesis and study of the antimicrobial activity of nifuroxazide derivatives. Microbiol. Indep. Res. J..

[13] Coxon G.D., Craig D., Corrales R.M., Vialla E., Gannoun-Zaki L., Kremer L. (2013). Synthesis, Antitubercular Activity and Mechanism of Resistance of Highly Effective Thiacetazone Analogues. PLoS ONE.

[14] Alafeefy A.M., Bakht M.A., Ganaie M.A., Ansarie M.N., El-Sayed N.N., Awaad A.S. (2015). Synthesis, analgesic, anti-inflammatory and anti-ulcerogenic activities of certain novel Schiff’s bases as fenamate isosteres. Bioorganic Med. Chem. Lett..

[15] Wang Y.Y., Xu F.Z., Zhu Y.Y., Song B., Luo D., Yu G., Chen S., Xue W., Wu J. (2018). Pyrazolo [3,4-d]pyrimidine derivatives containing a Schiff base moiety as potential antiviral agents. Bioorganic Med. Chem. Lett..

[16] Bonandi E., Christodoulou M.S., Fumagalli G., Perdicchia D., Rastelli G., Passarella D. (2017). The 1,2,3-triazole ring as a bioisostere in medicinal chemistry. Drug Discov. Today.

[17] Li H., Aneja R., Chaiken I. (2013). Click Chemistry in Peptide-Based Drug Design. Molecules.

[18] Angell Y.L., Burgess K. (2007). Peptidomimetics via copper-catalyzed azide–alkyne cycloadditions. Chem. Soc. Rev..

[19] Agalave S.G., Maujan S.R., Pore V.S. (2011). Click chemistry: 1,2,3-triazoles as pharmacophores. Chem. Asian J..

[20] Muller T., Bräse S. (2011). Click Chemistry Finds Its Way into Covalent Porous Organic Materials. Angew. Chemie Int. Ed..

[21] Smit F.J., Seldon R., Aucamp J., Jordaan A., Warner D.F., N’Da D.D. (2019). Synthesis and antimycobacterial activity of disubstituted benzyltriazoles. Med. Chem. Res..

[22] Aziz Ali A., Gogoi D., Chaliha A.K., Buragohain A.K., Trivedi P., Saikia P.J., Gehlot P.S., Kumar A., Chaturvedi V., Sarma D. (2017). Synthesis and biological evaluation of novel 1,2,3-triazole derivatives as anti-tubercular agents. Bioorganic Med. Chem. Lett..

[23] Zhang S., Xu Z., Gao C., Ren Q.C., Chang L., Lv Z.S., Feng L.S. (2017). Triazole derivatives and their anti-tubercular activity. Eur. J. Med. Chem..

[24] Zhang K., Wang P., Xuan L.-N., Fu X.-Y., Jing F., Li S., Liu Y.-M., Chen B.-Q. (2014). Synthesis and antitumor activities of novel hybrid molecules containing 1,3,4-oxadiazole and 1,3,4-thiadiazole bearing Schiff base moiety. Bioorganic Med. Chem. Lett..

[25] Aouad M. (2014). Synthesis, Characterization and Antimicrobial Evaluation of Some New Schiff, Mannich and Acetylenic Mannich Bases Incorporating a 1,2,4-Triazole Nucleus. Molecules.

[26] Aouad M.R. (2015). Efficient eco-friendly solvent-free click synthesis and antimicrobial evaluation of new fluorinated 1,2,3-triazoles and their conversion into schiff bases. J. Braz. Chem. Soc..

[27] Rezki N., Al-Yahyawi A., Bardaweel S., Al-Blewi F., Aouad M. (2015). Synthesis of Novel 2,5-Disubstituted-1,3,4-thiadiazoles Clubbed 1,2,4-Triazole, 1,3,4-Thiadiazole, 1,3,4-Oxadiazole and/or Schiff Base as Potential Antimicrobial and Antiproliferative Agents. Molecules.

[28] Almehmadi M.A., Aljuhani A., Alraqa S.Y., Ali I., Rezki N., Aouad M.R., Hagar M. (2021). Design, synthesis, DNA binding, modeling, anticancer studies and DFT calculations of Schiff bases tethering benzothiazole-1,2,3-triazole conjugates. J. Mol. Struct..

[29] Tanoli S.T., Ramzan M., Hassan A., Sadiq A., Jan M.S., Khan F.A., Ullah F., Ahmad H., Bibi M., Mahmood T. (2020). Design, synthesis and bioevaluation of tricyclic fused ring system as dual binding site acetylcholinesterase inhibitors. Int. J. Mol. Sci..

[30] Rezki N., Al-Sodies S.A., Aouad M.R., Bardaweel S., Messali M., El Ashry E., Ashry E.S.H.E. (2016). An eco-friendly ultrasound-assisted synthesis of novel fluorinated pyridinium salts-based hydrazones and antimicrobial and antitumor screening. Int. J. Mol. Sci..

[31] Aouad M.R., Messali M., Rezki N., Ali A.A.S., Lesimple A. (2015). Synthesis and characterization of some novel 1,2,4-triazoles, 1,3,4-thiadiazoles and Schiff bases incorporating imidazole moiety as potential antimicrobial agents. Acta Pharm..

[32] D’yakonov V.A., Finkelshtein E.S., Ibragimov A.G. (2007). Dzhemilev reaction for the synthesis of spiro[3.3]heptane and spiro[3.4]octanes. Tetrahedron Lett..

[33] Dheer D., Singh V., Shankar R. (2017). Medicinal attributes of 1,2,3-triazoles: Current developments. Bioorganic Chem..

[34] Wasilenko W.J., Palad A.J., Somers K.D., Blackmore P.F., Kohn E.C., Rhim J.S., Wright G.L., Schellhammer P.F. (1996). Effects of the calcium influx inhibitor carboxyamido-triazole on the proliferation and invasiveness of human prostate tumor cell lines. Int. J. Cancer.

[35] Allouche A. (2012). Software News and Updates Gabedit—A Graphical User Interface for Computational Chemistry Softwares. J. Comput. Chem..

[36] Kumar P., Kadyan K., Duhan M., Sindhu J., Singh V., Saharan B.S. (2017). Design, synthesis, conformational and molecular docking study of some novel acyl hydrazone based molecular hybrids as antimalarial and antimicrobial agents. Chem. Cent. J..

[37] Patorski P., Wyrzykiewicz E., Bartkowiak G. (2013). Synthesis and conformational assignment of N-(E)- stilbenyloxymethylenecarbonyl-substituted hydrazones of acetone and o-(m-and p-) chloro- (nitro-) benzaldehydes by means of 1H and 13C NMR spectroscopy. J. Spectrosc..

[38] Wyrzykiewicz E., Prukała D. (1998). New isomeric *N* -substituted hydrazones of 2-, 3- and 4-pyridinecarboxaldehydes. J. Heterocycl. Chem..

[39] Palla G., Predieri G., Domiano P., Vignali C., Turner W. (1986). Conformational behaviour and E/Z isomerization of N-acyl and N-aroylhydrazones. Tetrahedron.

[40] Chauhan N. (2020). Possible Drug Candidates for COVID-19 Possible Drug Candidates for COVID-19. ChemRxiv.

[41] Jin Z., Du X., Xu Y., Deng Y., Liu M., Zhao Y., Zhang B., Li X., Zhang L., Peng C. (2020). Structure of Mpro from COVID-19 virus and discovery of its inhibitors. Nature.

[42] DeLano W.L. (2004). PyMOL Reference Guide.

[43] Shawon J., Khan A.M., Rahman A., Hoque M.M., Khan M.A.K., Sarwar M.G., Halim M.A. (2018). Molecular Recognition of Azelaic Acid and Related Molecules with DNA Polymerase I Investigated by Molecular Modeling Calculations. Interdiscip. Sci. Comput. Life Sci..

[44] Wallace A.C., Laskowski R.A., Thornton J.M. (1995). Ligplot: A program to generate schematic diagrams of protein-ligand interactions. Protein Eng. Des. Sel..

[45] Alsafi M.A., Hughes D.L., Said M.A. (2020). First COVID-19 molecular docking with a chalcone-based compound: Synthesis, single-crystal structure and Hirshfeld surface analysis study. Acta Crystallogr. Sect. C Struct. Chem..

[46] Wang F., Chen C., Tan W., Yang K., Yang H. (2016). Structure of Main Protease from Human Coronavirus NL63: Insights for Wide Spectrum Anti-Coronavirus Drug Design. Sci. Rep..

[47] Liming Y., Yang C., Rao Z., Ren Z., Yan L., Zhang N., Guo Y., Yang C., Lou Z., Rao Z. (2013). The newly emerged SARS-Like coronavirus HCoV-EMC also has an “Achilles’’ heel: Current effective inhibitor targeting a 3C-like protease. Protein Cell.

[48] Xue X., Yu H., Yang H., Xue F., Wu Z., Shen W., Li J., Zhou Z., Ding Y., Zhao Q. (2008). Structures of Two Coronavirus Main Proteases: Implications for Substrate Binding and Antiviral Drug Design Downloaded from. J. Virol..

[49] Yang H., Yang M., Ding Y., Liu Y., Lou Z., Zhou Z., Sun L., Mo L., Ye S., Pang H. (2003). The crystal structures of severe acute respiratory syndrome virus main protease and its complex with an inhibitor. Proc. Natl. Acad. Sci. USA.

[50] Anand K., Palm G.J., Mesters J.R., Siddell S.G., Ziebuhr J., Hilgenfeld R. (2002). Structure of coronavirus main proteinase reveals combination of a chymotrypsin fold with an extra α-helical domain. EMBO J..

[51] Bursulaya B.D., Totrov M., Abagyan R., Brooks C.L. (2003). Comparative study of several algorithms for flexible ligand docking. J. Comput. Aided. Mol. Des..

[52] De Magalhães C.S., Barbosa H.J.C.C., Dardenne L.E. (2004). A Genetic Algorithm for the Ligand-Protein Docking Problem. Genet. Mol. Biol..

[53] Sterling T., Irwin J.J. (2015). ZINC 15-Ligand Discovery for Everyone. J. Chem. Inf. Model..

[54] Ton A.T., Gentile F., Hsing M., Ban F., Cherkasov A., Sterling T., Irwin J.J. (2020). Rapid Identification of Potential Inhibitors of SARS-CoV-2 Main Protease by Deep Docking of 1.3 Billion Compounds. Mol. Inform..

[55] Hall D.C., Ji H.F. (2020). A search for medications to treat COVID-19 via in silico molecular docking models of the SARS-CoV-2 spike glycoprotein and 3CL protease. Travel Med. Infect. Dis..

[56] Wang Q., Zhou Y., Rychahou P., Fan T.W.M., Lane A.N., Weiss H.L., Evers B.M. (2017). Ketogenesis contributes to intestinal cell differentiation. Cell Death Differ..

[57] Kumar S., Stecher G., Li M., Knyaz C., Tamura K. (2018). MEGA X: Molecular evolutionary genetics analysis across computing platforms. Mol. Biol. Evol..

[58] Crooks G.E., Hon G., Chandonia J.M., Brenner S.E. (2004). WebLogo: A sequence logo generator. Genome Res..

[59] Schneider T.D., Stephens R.M. (1990). Sequence logos: A new way to display consensus sequences. Nucleic Acids Res..

[60] Ortega J.T., Serrano M.L., Pujol F.H., Rangel H.R. (2020). Unrevealing sequence and structural features of novel coronavirus using in silico approaches: The main protease as molecular target. EXCLI J..

