# Supplementary material for: New 1,2,3-Triazole Scaffold Schiff Bases as Potential Anti-COVID-19: Design, Synthesis, DFT-Molecular Docking, and Cytotoxicity Aspects
Source: Vaccines (Basel). 2021 Sep 11;9(9):1012. doi: 10.3390/vaccines9091012 (PMC8472185; doi:10.3390/vaccines9091012)
Supplement: Supplementary file 1 [file vaccines-09-01012-s001.zip › vaccines-1341750-supplementary.pdf]

Supporting Information

New 1,2,3-triazole Scaffold Schiff Bases as Potential Anti-COVID-19; Design, Synthesis, DFT-Molecular Docking Aspects, and Cytotoxicity Computer-aided Assessment

Musa A. Said,[1,*] Daoud J. O. Khan,[1] Fawzia F Al-blewi,[1] Nadia S. Al-Kaff [2], Adeeb A. Ali,[1] Nadjet Rezki,[1] Mohamed Reda Aouad,[1,*] Mohamed Hagar[3,4]

[1]Department of Chemistry, College of Science, Taibah University, Al-Madinah Al-Munawarah 30002, Saudi Arabia

[2]Department of Biology, College of Science, Taibah University, Al-Madinah Al-Munawarah 30002, Saudi Arabia

[3]Chemistry Department, College of Sciences, Yanbu, Taibah University, Yanbu, 30799, Saudi Arabia

[4]Chemistry Department, Faculty of Science, Alexandria University, Alexandria 21321, Egypt

* Correspondence: authors at: masaid@taibahu.edu.sa, maouad@taibahu.edu.sa

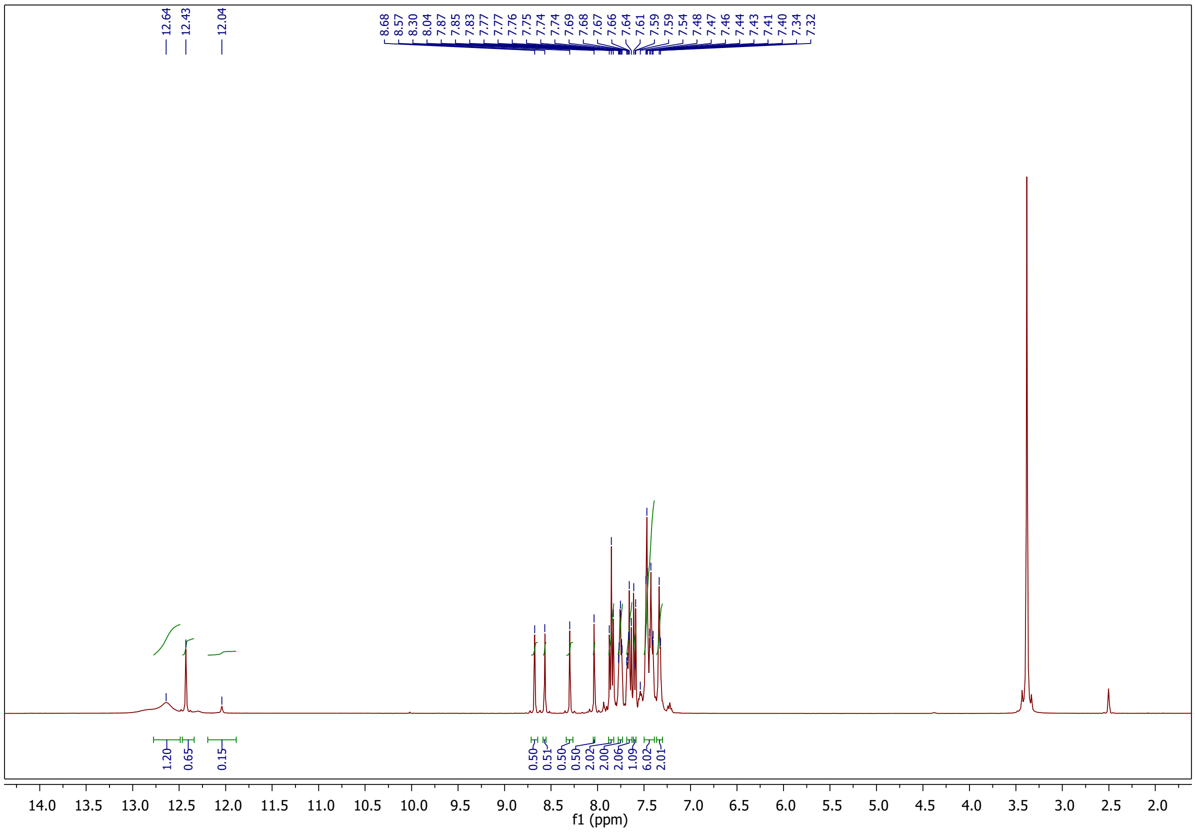

Figure S1. ¹H NMR of Compound **5a**

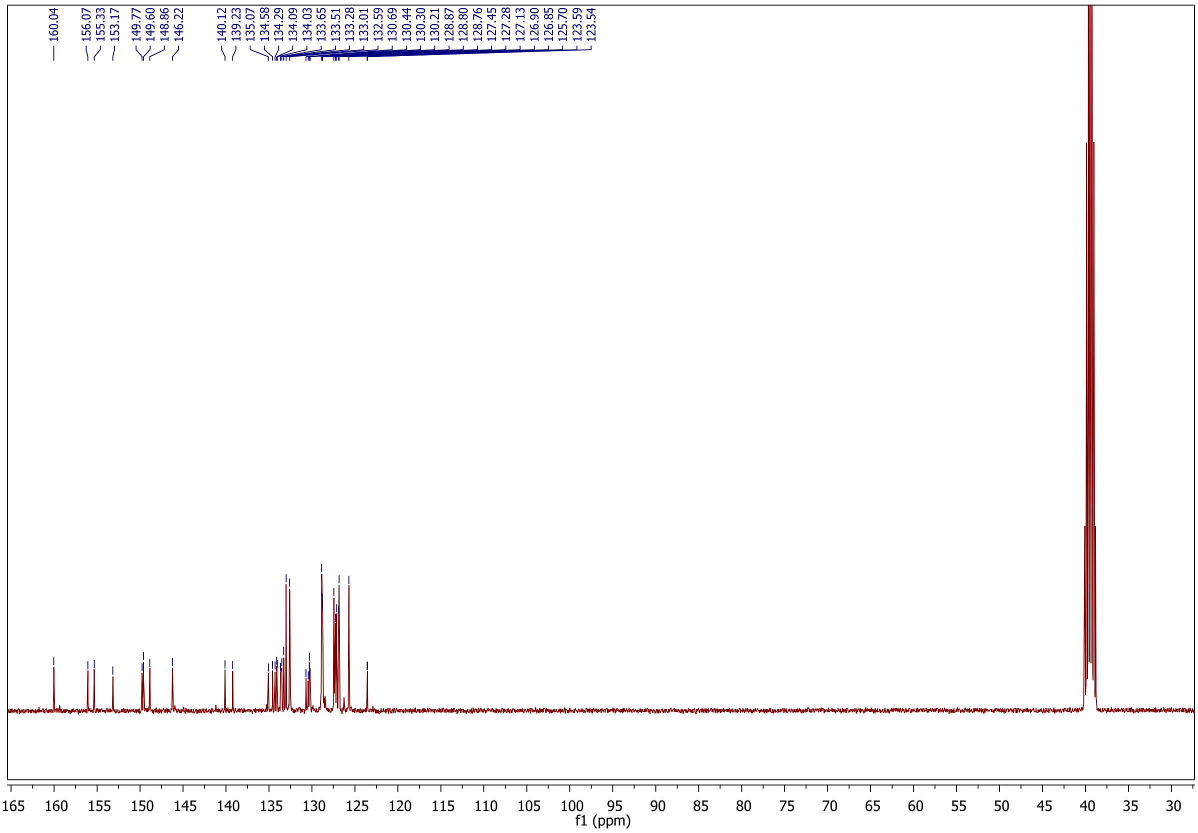

Figure S2. ¹³C NMR of Compound **5a**

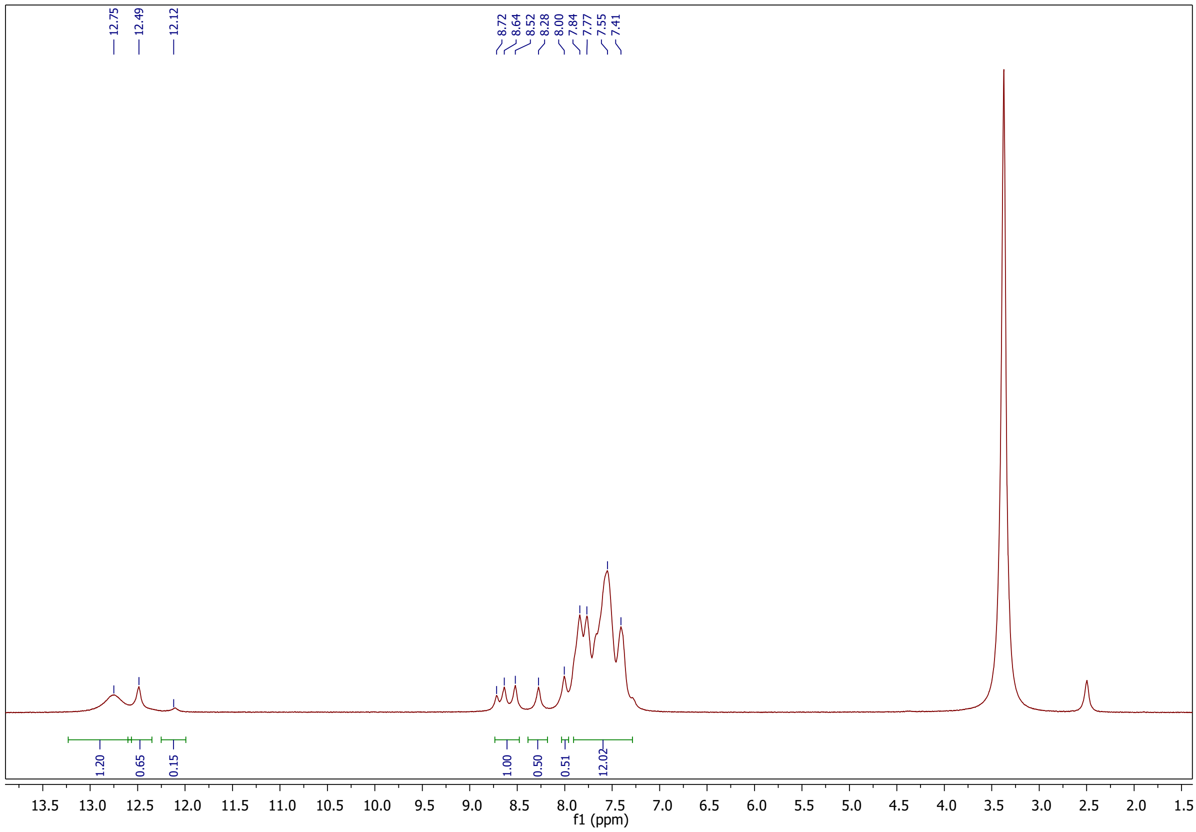

Figure S3. ^{1}H NMR of Compound **5b**

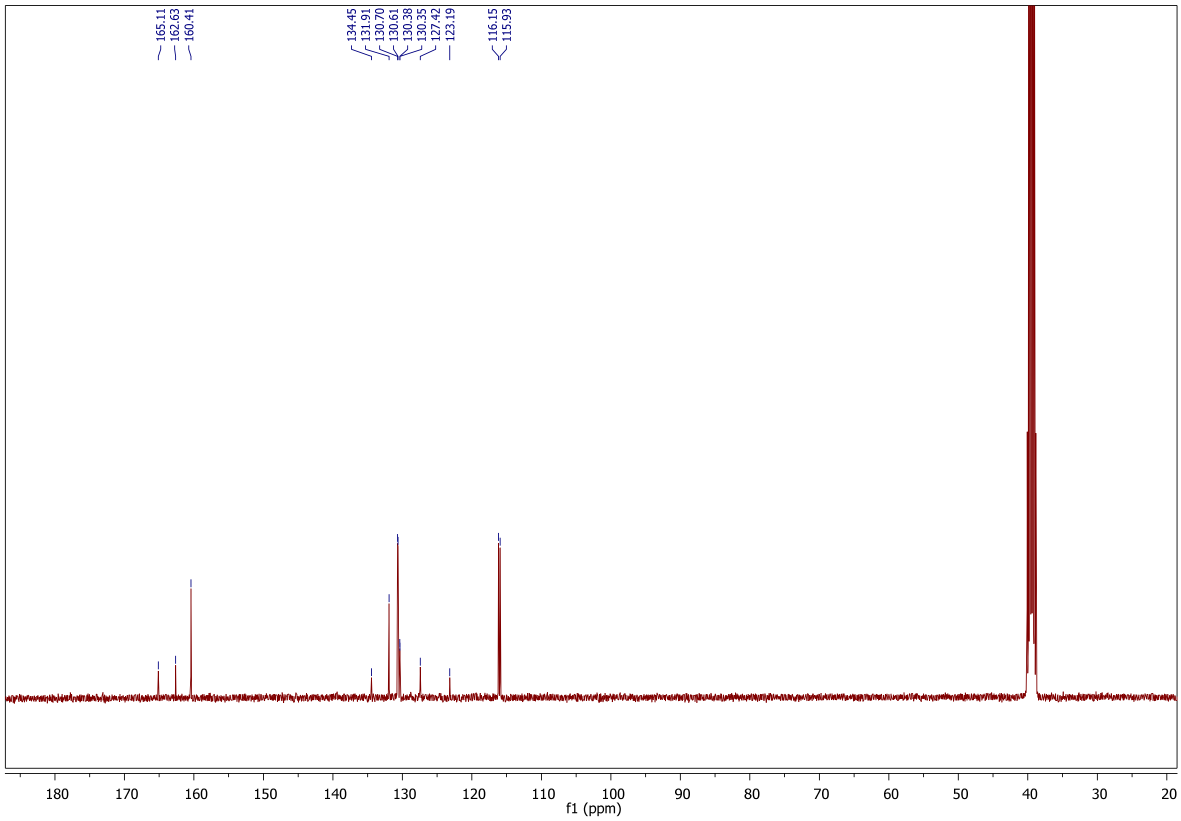

Figure S4. ^{13}C NMR of Compound **5b**

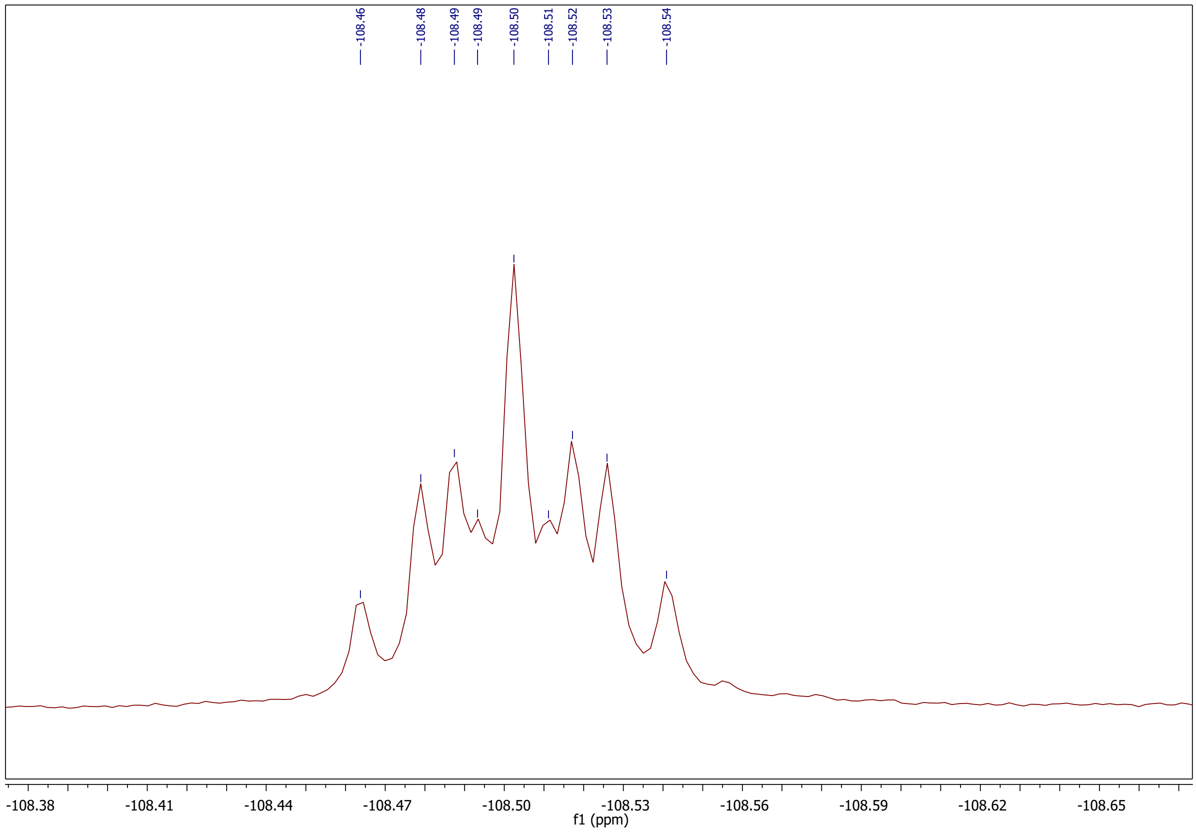

Figure S5. ^{19}F NMR of Compound **5b**

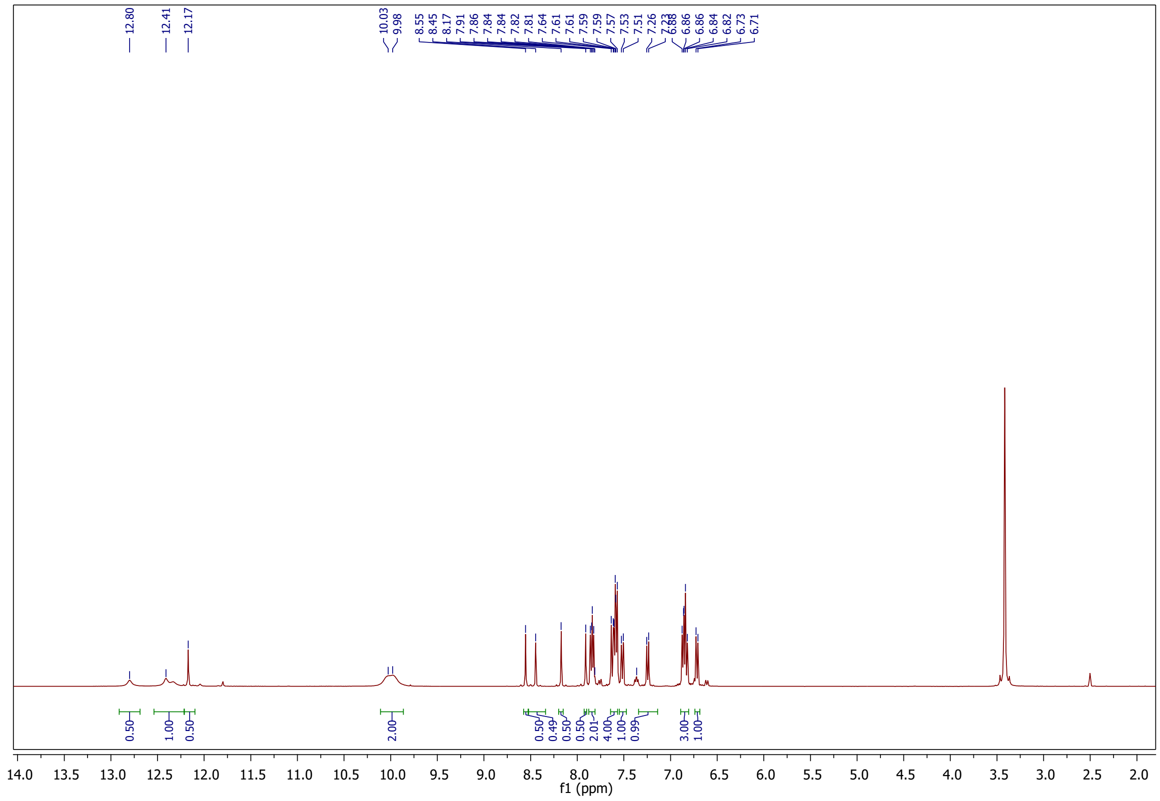

Figure S6. ^{1}H NMR of Compound **5e**

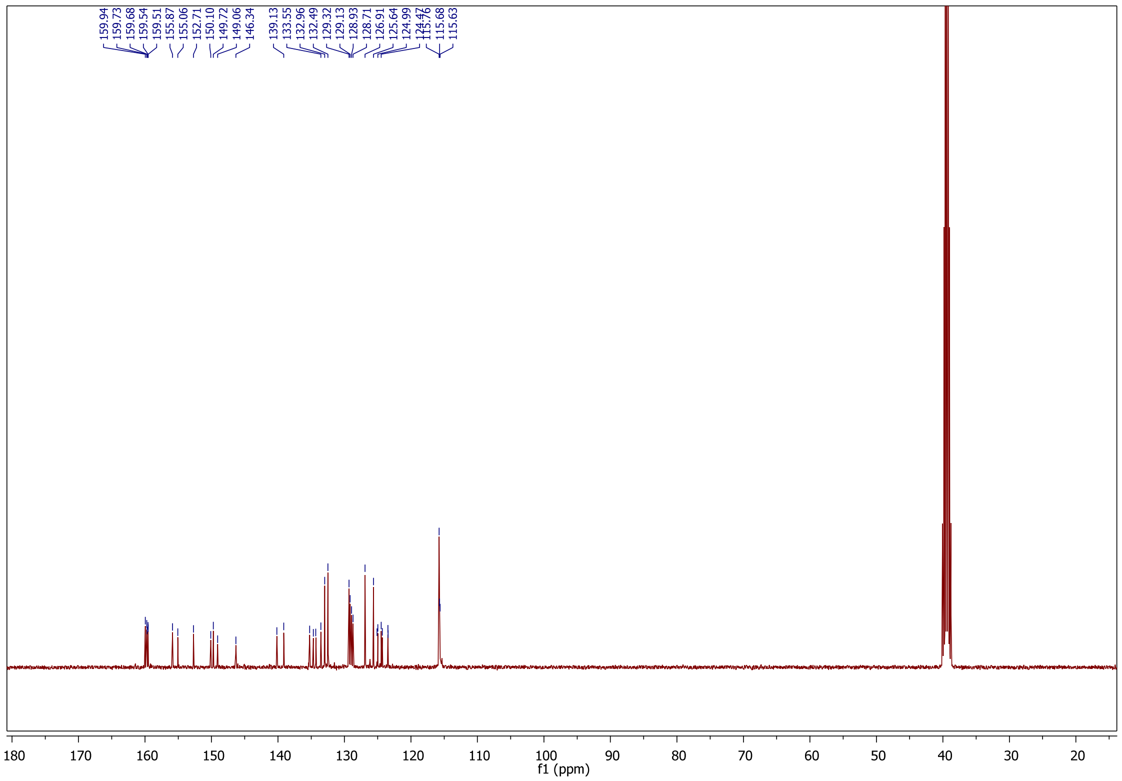

Figure S7. ^{13}C NMR of Compound **5e**

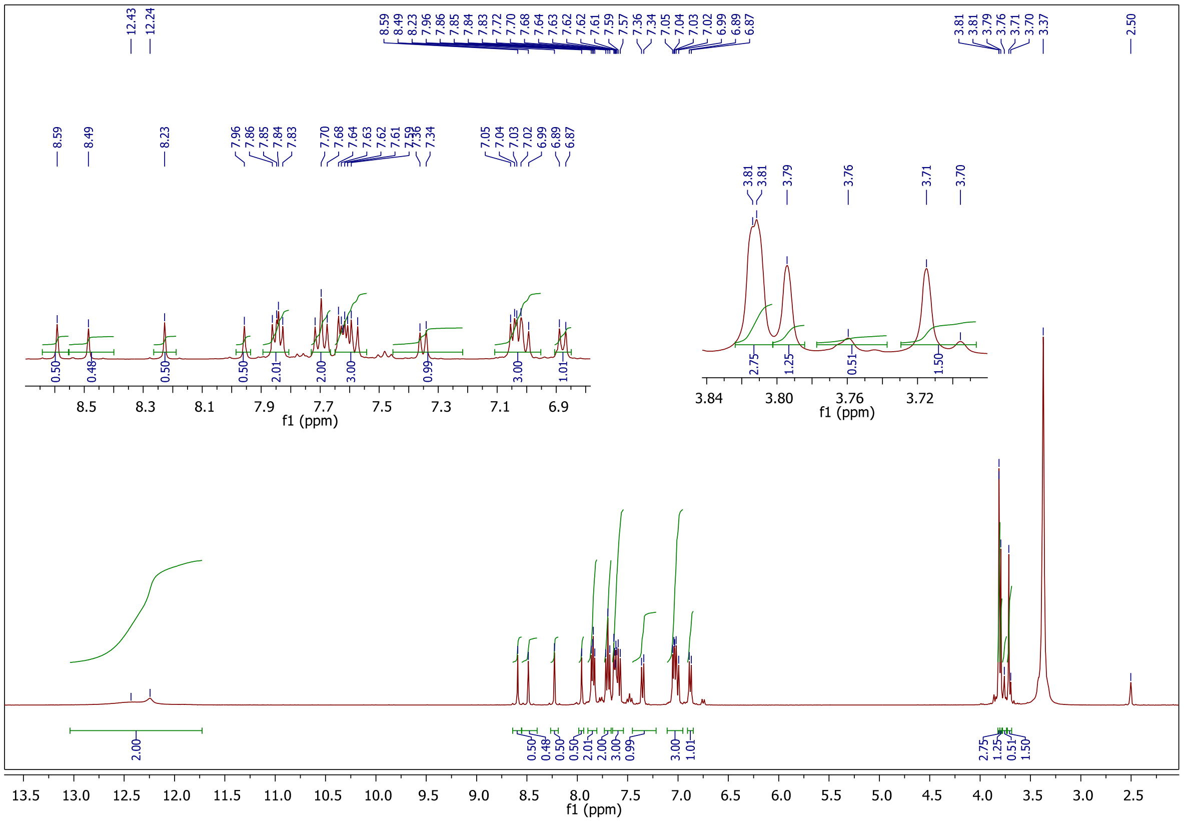

Figure S8. ^{1}H NMR of Compound **5f**

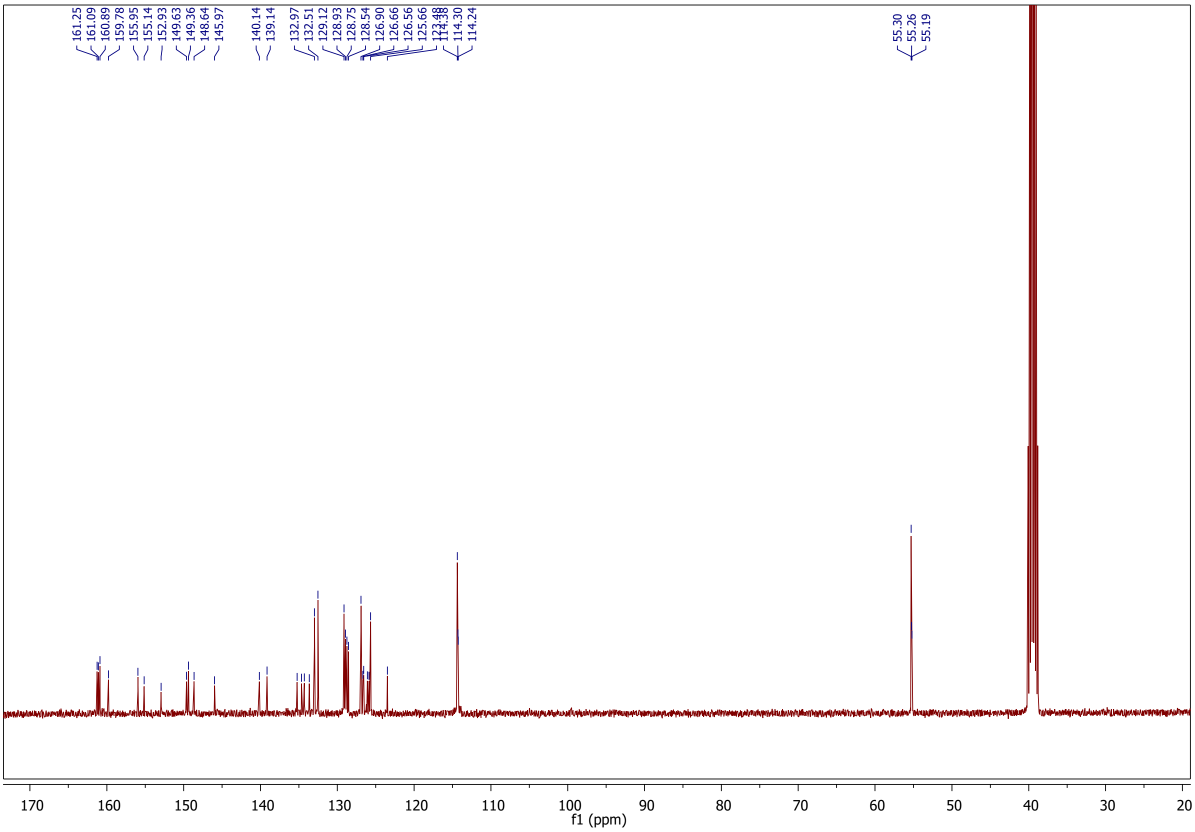

Figure S9. ^{13}C NMR of Compound **5f.**

Schiff Base	Amino acids																															
Residues based on COVID-19	T24		T25		T26		L27		H41		T45		S46		M49		Y54		F140		L141		N142		G143		S144		C145		H163	
Legand name	H	Y	H	Y	H	Y	H	Y	H	Y	H	Y	H	Y	H	Y	H	Y	H	Y	H	Y	H	Y	H	Y	H	Y	H	Y	H	Y
5a	0	0	0	0	0	1	0	0	0	1	0	0	0	0	0	1	0	0	0	1	0	1	1	0	0	0	0	0	0	1	0	0
5b	0	0	0	1	0	0	0	1	0	1	0	0	0	0	0	0	0	0	0	0	0	0	0	1	0	0	0	0	0	0	0	0
5c	0	0	0	1	0	0	0	1	0	1	0	0	0	0	0	1	0	0	0	0	0	0	0	1	0	1	0	0	0	0	0	0
5d	0	0	0	1	0	1	0	0	0	0	0	0	0	0	0	1	0	0	0	0	0	0	0	1	1	0	0	0	0	1	0	0
5e	0	0	0	1	0	0	0	0	0	0	0	0	0	0	0	0	0	0	0	1	1	0	0	1	1	0	0	0	0	1	0	0
5f	0	0	0	1	0	1	0	1	0	1	0	0	0	0	0	1	0	1	0	0	0	1	0	1	0	0	0	0	0	0	0	0
5g	0	0	0	0	0	0	0	0	1	0	0	0	0	0	0	0	0	0	0	0	1	0	0	1	0	0	0	0	0	1	1	0
Total	0	0	0	5	0	3	0	3	1	4	0	0	0	0	0	4	0	1	0	2	2	2	1	6	2	1	0	0	0	4	1	0
%	0	0	0	#	0	#	0	#	#	#	0	0	0	0	0	#	0	#	0	#	#	#	#	#	#	#	0	0	0	#	#	0

Schiff Base	Amino acids																											
Residues based on COVID-19	H164		M165		E166		L167		P168		H172		F185		D187		R188		Q189		T190		A191		Q192			
Legand name	H	Y	H	Y	H	Y	H	Y	H	Y	H	Y	H	Y	H	Y	H	Y	H	Y	H	Y	H	Y	H	Y		
5a	0	1	0	1	1	0	0	1	0	1	0	0	0	0	0	0	0	1	1	0	0	1	0	0	0	1	3	#
5b	0	1	0	1	0	1	0	0	0	0	0	0	0	0	0	0	0	1	1	0	0	0	0	0	0	0	1	7
5c	0	1	0	1	1	0	0	1	0	1	0	0	0	0	0	1	0	0	0	1	0	0	0	0	0	0	1	#
5d	1	0	0	1	0	1	0	0	0	0	0	0	0	0	0	0	0	1	0	1	0	0	0	0	0	0	2	9
5e	0	0	0	1	0	1	0	0	0	0	0	0	0	0	0	0	1	0	1	0	1	0	0	0	0	0	5	6
5f	0	0	0	1	1	0	0	1	0	1	0	0	0	0	0	1	0	1	0	1	0	1	0	0	0	0	1	#
5g	0	1	0	1	2	0	0	1	0	0	0	0	0	0	0	1	0	1	0	1	0	0	0	0	0	0	4	9
Total	1	4	0	7	5	3	0	4	0	3	0	0	0	0	0	3	1	5	3	4	1	2	0	0	0	1		
%	#	57	0	100	71	#	0	#	0	#	0	0	0	0	0	#	#	#	#	#	#	#	0	0	0	#		

Approved Medicine	Amino acids																															
Residues based on COVID-19	T24		T25		T26		L27		H41		T45		S46		M49		Y54		F140		L141		N142		G143		S144		C145		H163	
Legand name	H	Y	H	Y	H	Y	H	Y	H	Y	H	Y	H	Y	H	Y	H	Y	H	Y	H	Y	H	Y	H	Y	H	Y	H	Y	H	Y
Hydroxychloroquine	0	0	0	0	0	0	0	0	0	1	0	0	0	0	0	0	0	0	0	1	0	1	0	1	0	0	1	0	0	0	0	1
Favipiravir	0	0	0	0	0	0	0	0	1	0	0	0	0	0	0	0	1	0	0	0	0	0	0	0	0	0	0	0	0	0	0	0
Ribavirin	0	0	0	0	0	0	0	0	0	1	0	0	0	0	0	1	0	0	1	0	1	0	0	1	0	0	1	0	0	1	1	0
Amodiaquine	0	0	0	0	0	0	0	0	0	1	0	0	0	0	0	1	0	1	0	0	0	0	0	0	0	0	0	0	0	1	0	1
2'-Deoxy-2'-fluorocytidine	0	0	0	0	0	0	0	0	0	1	0	0	0	0	0	0	0	0	1	0	0	1	0	1	0	0	0	0	0	1	1	0
Remdesivir	0	0	0	1	3	0	0	1	0	1	0	0	1	0	0	1	0	0	0	0	0	0	0	1	0	1	0	0	0	1	0	0
N3	0	1	0	1	0	0	0	0	1	0	0	1	0	1	0	1	1	0	0	0	0	0	0	1	1	0	0	0	0	1	0	0
Total	0	1	0	2	3	0	0	1	2	5	0	1	1	1	0	4	2	1	2	1	1	2	0	5	1	1	2	0	0	5	2	2
%	0	14	0	#	#	0	0	#	#	#	0	#	#	#	0	#	#	#	#	#	#	#	0	#	#	#	#	0	0	#	#	#

Approved Medicine	Amino acids																											
Residues based on COVID-19	H164		M165		E166		L167		P168		H172		F185		D187		R188		Q189		T190		A191		Q192			
Legand name	H	Y	H	Y	H	Y	H	Y	H	Y	H	Y	H	Y	H	Y	H	Y	H	Y	H	Y	H	Y	H	Y		
Hydroxychloroquine	0	1	0	1	0	1	0	0	0	0	0	0	0	0	0	1	0	1	0	1	0	0	0	0	0	0	1	#
Favipiravir	0	0	0	1	0	0	0	0	0	0	0	0	0	0	0	1	0	1	0	1	0	0	0	0	0	0	2	4
Ribavirin	2	0	0	1	1	1	0	0	0	0	0	0	0	0	0	0	0	0	1	0	0	0	0	0	0	0	8	5
Amodiaquine	0	1	0	1	0	0	0	0	0	0	0	0	0	0	0	1	1	0	0	1	0	0	0	0	0	0	1	#
2'-Deoxy-2'-fluorocytidine	1	0	0	1	0	1	0	0	0	0	0	0	0	0	0	0	0	0	0	0	0	0	0	0	0	0	3	6
Remdesivir	0	1	0	1	1	0	0	0	0	0	0	0	0	0	0	1	0	1	1	0	0	0	0	0	0	0	6	#
N3	1	0	0	1	0	1	0	0	0	1	0	0	0	0	0	1	0	0	0	1	0	1	0	0	0	0	4	#
Total	4	3	0	7	2	4	0	0	0	1	0	0	0	0	0	5	1	3	2	4	0	1	0	0	0	0		
%	#	43	0	100	29	#	0	0	0	#	0	0	0	0	0	#	#	#	#	#	0	#	0	0	0	0		

Conserved amino acid

Sheet1

Figure S10. Ligand N3 was the only one that showed interaction with G143.
